# MGA directly recruits SETDB1/ATF7IP for histone H3K9me3 mark on meiosis-related genes in mouse embryonic stem cells

**DOI:** 10.1016/j.isci.2025.113059

**Published:** 2025-07-05

**Authors:** Kousuke Uranishi, Masataka Hirasaki, Masazumi Nishimoto, Robert J. Klose, Akihiko Okuda, Ayumu Suzuki

**Affiliations:** 1 Division of Biomedical Sciences, Research Center for Genomic Medicine, Saitama Medical University, 1397-1 Yamane Hidaka, Saitama 350-1241, Japan; 2 Department of Clinical Cancer Genomics, International Medical Center, Saitama Medical University, 1397-1 Yamane Hidaka, Saitama 350-1241, Japan; 3 Biomedical Research Center, Saitama Medical University, 38 Morohongo, Moroyama-machi, Iruma-gun, Saitama 350-0495, Japan; 4 Department of Biochemistry, University of Oxford, Oxford OX1 3QU, UK; 5 Laboratory Animal Resource Center in Trans-Border Medical Research Center, Institute of Medicine, University of Tsukuba, 1-1-1 Tennoudai, Itsukuba, Ibaraki 305-8575, Japan

**Keywords:** Biochemistry, Cell biology, Stem cells research

## Abstract

Polycomb repressive complex 1.6 (PRC1.6), one of the PRC1 subtypes, plays crucial roles in preventing the ectopic expression of meiosis-related genes in mouse embryonic stem cells (ESCs). In addition to the histone modifications H2AK119ub1 and H3K27me3 that are deposited by PRC1 and PRC2, respectively, many meiosis-related genes bear the trimethylated lysine 9 of histone H3 (H3K9me3) mark in ESCs. However, the precise molecular mechanisms that deposit this mark on these genes in ESCs remain unknown. Here, we demonstrated that MGA, a scaffolding component of PRC1.6, is directly involved in recruiting SETDB1, an enzyme that catalyzes this histone modification, via its interaction with ATF7IP. Thus, our findings indicate that MGA plays a dual role, first being central in establishing a PRC1/PRC2-dependent repressive state by contributing to the construction of PRC1.6 as a scaffolding component, and then inducing a more robustly repressed state by recruiting the STEDB1/ATF7IP complex for H3K9me3 modification.

## Highlights

• ATF7IP/SETDB1 complex acts in H3K9 trimethylation of meiosis-related genes in ESCs
• KAP1 does not help recruit ATF7IP/SETDB1 complex on meiosis-related genes in ESCs
• MGA, a scaffolding component of PRC1.6, directly recruits ATF7IP/SETDB1 complex
• MGA and ATF7IP utilize FAM and FNIII domains, respectively, for their interaction

## Graphical abstract

**Figure:**
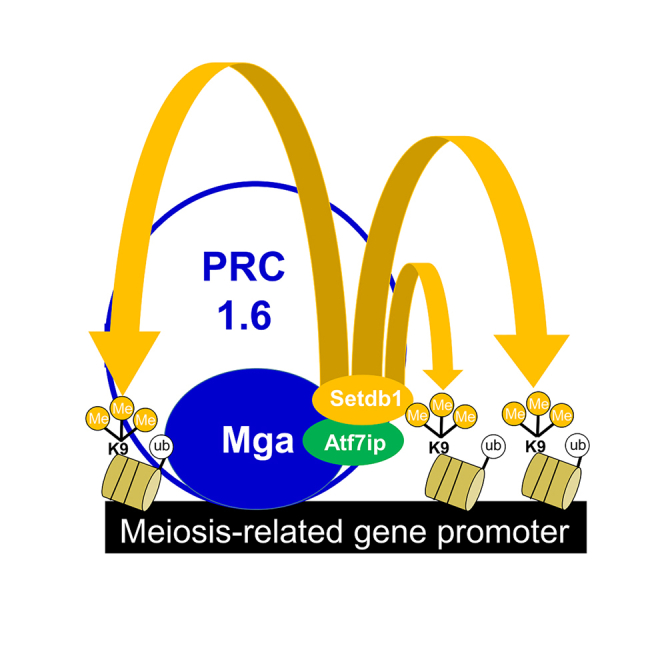

## Introduction

During mammalian development, cell fate decisions and cell maturation are largely dictated by dynamic changes in gene expression networks. These changes are mainly controlled by complex interplay between transcriptional activators/repressors and epigenetic regulators.^1^^,^^2^^,^^3^ Epigenetic regulators are not necessarily linked to transcriptional activation, but many of them are involved in the transcriptional repression of genes whose expression is unnecessary for given cells. Such transcriptional repression alongside the transcriptional activation of necessary genes is extremely important for given cells because the ectopic expression of non-physiological genes is usually associated with various types of abnormalities, such as differentiation failure, cell death, and tumorigenesis.^4^^,^^5^^,^^6^

Germline genes, which are essential for gametogenesis, are one example of genes subjected to dynamic regulation during embryonic development.^7^^,^^8^ However, the way in which the expression of germline genes is regulated is distinct from that of non-germline genes in certain respects. Similarly to non-germline genes, germline genes in preimplantation embryos are under less robust repression with no DNA methylation, but are subjected to extensive DNA methylation around the implantation stage. However, germline genes acquire DNA methylation within CpG island promoters where DNA methylation is usually not present irrespective of the expression state of non-germline genes.^9^^,^^10^ While this DNA methylation is erased after the differentiation of primordial germ cells from epiblast cells, germline genes retain a hypermethylated state of CpG promoter in non-germ cell lineages. An additional notable characteristic of germline genes regarding epigenetic modifications is that many of them bear the trimethylated lysine 9 of histone H3 (H3K9me3) mark in addition to ubiquitinated lysine 119 of histone H2A (H2AK119ub1) and trimethylated lysine 27 of histone H3 (H3K27me3) in naive embryonic stem cells (ESCs),^11^ which are faithfully represented *in vitro* counterparts of pluripotent cells in preimplantation embryos.^12^

Previously, we and other groups reported that polycomb repressive complex 1.6 (PRC1.6), one of the noncanonical PRC1 complexes, plays a central role in repressing a substantial number of germline/meiosis-related genes in mouse ESCs.^13^^,^^14^^,^^15^^,^^16^^,^^17^^,^^18^^,^^19^^,^^20^^,^^21^^,^^22^ Similar to other PRC1 complexes, PRC1.6 comprises many proteins, including components shared among multiple types of PRC1 (e.g., RING1A or B and RYBP) and PRC1.6-specific components (e.g., PCGF6 and L3MBTL2).^23^ Notably, MGA and E2F, both of which are also PRC1.6-specific components, exhibit intrinsic DNA binding activity by interacting with MAX and DP-1, respectively.^24^^,^^25^ We previously demonstrated that deletion of the basic-helix-loop-helix domain (bHLH), one of two DNA binding domains of MGA, results in strong reduction in DNA methylation levels in epiblast-like cells (EpiLCs), *in vitro* counterparts of post-implantation epiblast cells,^26^ whereas only slight reduction is observed upon disruption of the *Pcgf6* gene.^11^ Our data have also demonstrated that loss of PCGF6 and, in particular, deletion of the bHLH domain of MGA induces significant reduction in H3K9m3 levels in both ESCs and EpiLCs.^11^ These results indicate that PRC1.6 components, in particular MGA, are involved in DNA methylation and H3K9me3 modification on germline genes. However, the molecular mechanisms by which germline genes acquire these epigenetic modifications in a PRC1.6 component-dependent manner remain elusive.

Here, we demonstrated that MGA is directly involved in recruiting SETDB1 (also known as ESET), an enzyme that catalyzes histone H3K9m3 modification,^27^^,^^28^ via its interaction with ATF7IP (also known as MCAF1 or AM), a binding partner for SETDB1.^29^^,^^30^ This leads to the establishment of a chromatin state that is predisposed to DNA methylation for robust repression.

## Results

### Clustering of genomic MGA binding sites according to their interaction with PCGF6 and factors related to histone H3K9 methylation

**Figure 1:**
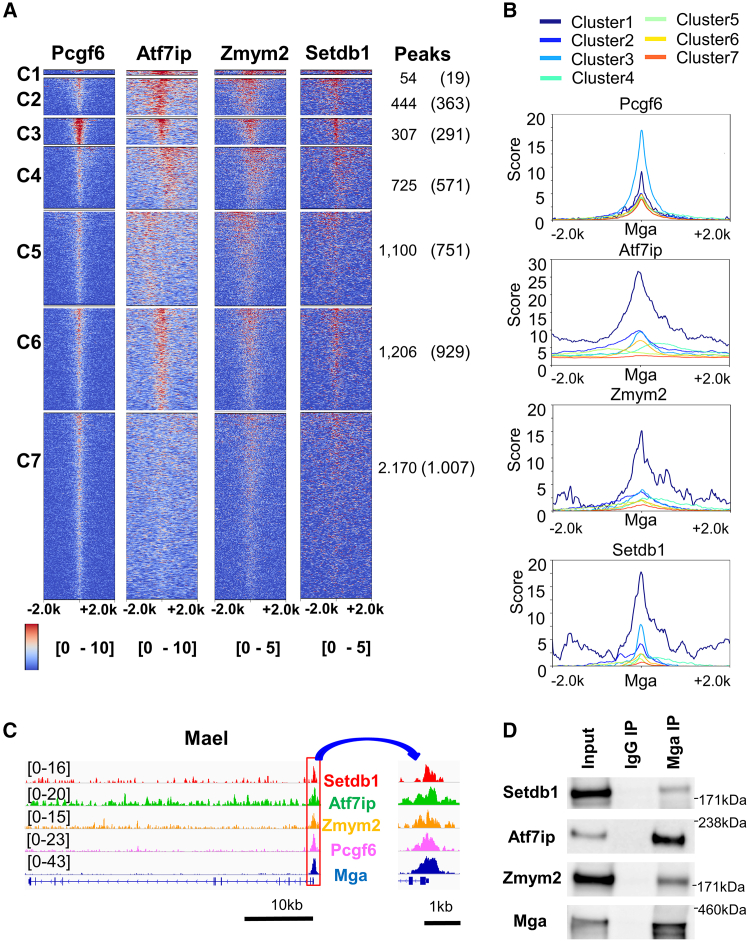
Classification of genomic MGA binding sites according to the interaction of PCGF6 and H3K9me3-related factors (A) Heatmaps of PCGF6, ATF7IP, ZMYM2, and SETDB1 on genomic MGA binding sites were partitioned into seven clusters using a k-means algorithm. (B) Metaplots of PCGF6, ATF7IP, ZMYM2, and SETDB1 over genomic MGA binding sites. Seven cluster gene groups were individually used to obtain data on these factors. (C) Genomic snapshots showing densities of SETDB1, ATF7IP, ZMYM2, PCGF6, and MGA over the *Mael* gene in ESCs. Right panel shows an enlarged image of the promoter region of the gene indicated by a red box in the left panel. (D) Coimmunoprecipitation analyses were performed with anti-MGA antibody using nuclear extracts from wild-type ESCs. IgG from non-immune rabbits was used as a negative control for immunoprecipitation.

To explore the relationship between MGA and H3K9me3 mark, we inspected the levels of enrichment of factors related to this mark (ZMYM2, ATF7IP, SETDB1, and KAP1, also known as TRIM28 or TIF1B)^27^^,^^28^^,^^29^^,^^30^^,^^31^^,^^32^^,^^33^^,^^34^ on genomic MGA binding sites using publicly available ChIP-sequence data (datasets used are listed in the key resources table in STAR Methods). These analyses revealed that, like PCGF6 (a central component of PRC1.6),^23^ factors related to histone H3K9 methylation (ZMYM2, ATF7IP, and SETDB1) were significantly enriched at genomic sites bound to MGA, which binds to the genome either as a component of PRC1.6 or independently of this complex (Figure S1A). However, notably, KAP1, which extensively colocalizes with ATF7IP and SETDB1 on endogenous retroviruses (ERVs) with the H3K9me3 modification mark,^31^^,^^32^ was not enriched at these genomic sites, indicating that the ATF7IP/SETDB1 complex is recruited in distinct ways between ERVs and MGA-bound genomic sites. Next, we subdivided MGA-bound genomic sites according to the levels of accumulation of four factors (PCGF6, ZMYM2, ATF7IP, and SETDB1) (Figure 1A). These procedures yielded seven clusters (genes included in each cluster are listed in Table S2) in which cluster 3 and cluster 1 were characterized as the most and second most PCGF6-enriched MGA binding sites. As for histone H3K9 methylation-related factors, cluster 1 showed the strongest enrichment of all of these factors; these factors, in particular SETDB1, also showed fairly high accumulation in cluster 3 (Figures 1A and 1B). Although cluster 1 and cluster 3 genes were apparently only minor populations among all MGA-bound genes, both clusters, in particular cluster 3, represent major populations among presumed PRC1.6-bound genomic sites due to the association with PCGF6 as well as MGA (Figure S2). Because we and others previously demonstrated the strong link between PCGF6-containing PRC1.6 and meiosis-related genes in ESCs,^17^^,^^18^^,^^19^ we investigated whether meiosis-related genes were enriched in cluster 1, cluster 3, or both using gene ontology (GO) analyses (Figure S3). These analyses indicated an abundance of meiosis-related genes in cluster 3. However, most other clusters including cluster 1 were not suggested to be linked to terms related to gametogenesis or meiosis, although a rather weak link between cluster 7 genes and spermatogenesis was suggested by the analysis. These results indicated that, unlike in cluster 3, meiosis-related genes are not enriched in cluster 1. Figure 1C shows representative snapshots of the ChIP-sequence data of *Mael*, which is one of the meiosis-related genes classified into cluster 3, showing that all of these four factors (i.e., SETDB1, ATF7IP, ZMYM2, and PCGF6) were colocalized with MGA around their transcription start sites (TSSs). This implies that these factors form a large complex on MGA binding sites of the genome. Consistent with this assumption, coimmunoprecipitation analyses revealed that ZMYM2, ATF7IP, and SETDB1 were all coimmunoprecipitated with antibody against MGA (Figure 1D) using nuclear extract from ESCs; moreover, the analyses with DNase and RNase indicated that neither DNA nor RNA was required for these protein-protein interactions (Figure S4).

### ATF7IP and SETDB1, but not ZMYM2, are involved in deposition of H3K9me3 modification on meiosis-related genes

**Figure 2:**
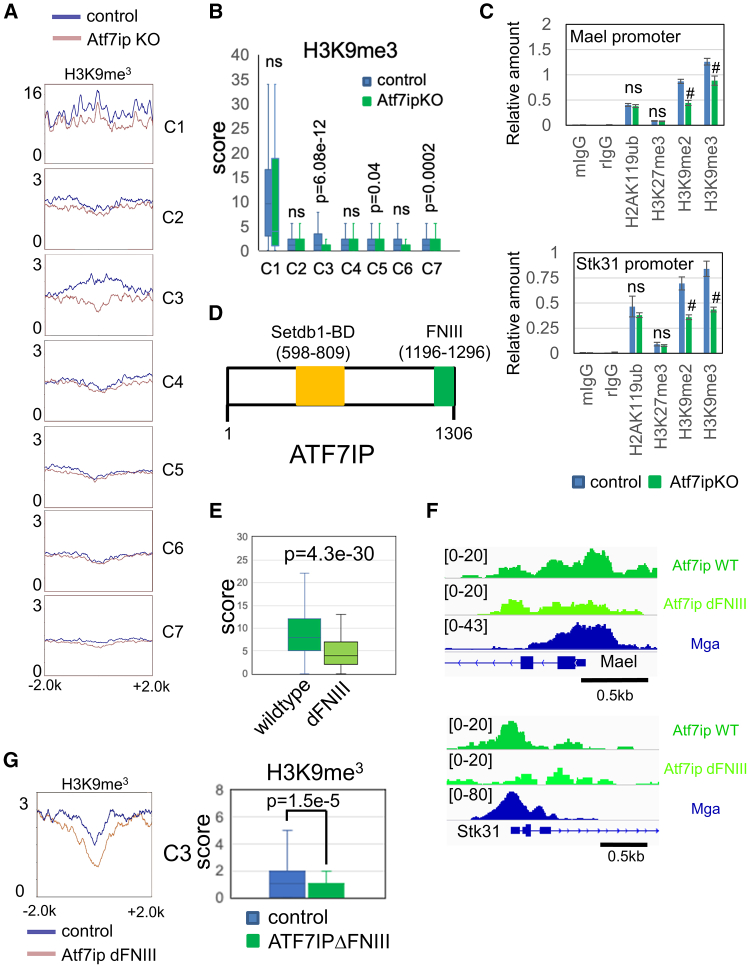
ATF7IP-dependent deposition of H3K9me3 mark on cluster 1 and 3 genes (A) Comparison of the effect of disruption of the *Atf7ip* gene on H3K9me3 levels among seven MGA binding site-containing gene cluster sets in ESCs. An image covering a broader region (−10k to +10k) is provided in Figure S6. (B) Quantitative analysis of the difference in H3K9me3 levels due to *Atf7ip* gene disruption in each gene cluster using a boxplot. Statistical significance was assessed using two-tailed unpaired Welch’s t test. ns, *p* > 0.05. (C) ChIP-qPCR analyses of H2AK119ub, H3K27me3, H3K9me2, and H3K9me3 on *Mael* and *Stk31* gene promoters using wild-type and *Atf7ip*-null ESCs. Data represent mean ± SD of three independent experiments. Two-tailed unpaired Welch’s t test was conducted to examine statistical significance. #, *p* < 0.05; ns, *p* > 0.05. (D) Schema of two functional domains of ATF7IP. (E) Quantitative analysis of the difference in the levels of binding to cluster 3 genes between wild-type ATF7IP and its mutant lacking the FNIII domain using a boxplot. These data were obtained using publicly available ChIP-seq data generated using an anti-Flag antibody (accession number PRJNA664286)^34^ in which both wild-type and mutant ATF7IPs are expressed as Flag-tagged proteins in *Atf7ip*-null ESCs. Statistical significance was assessed as in (B). (F) Genomic snapshots showing densities of wild-type ATF7IP, ATF7IP with no FNIII domain, and MGA over *Mael* and *Stk31* genes in ESCs. These data were obtained with the same ChIP-seq data used in (E). (G) Effect of deletion of FNIII domain from ATF7IP on H3K9me3 levels of cluster 3 genes in ESCs. Left and right panels show metagene plots of H3K9me3 on cluster 3 genes in wild-type ESCs and those expressing mutant ATF7IP lacking its FNIII domain and a boxplot showing the quantitative difference of H3K9me3 levels due to the loss of the FNIII domain from ATF7IP in ESCs. Statistical significance was assessed as in (B).

Next, we examined the consequence of the disruption of *Atf7ip*, *Zmym2*, and *Setdb1* genes on H3K9me3 repressive histone marks on MGA target genes. Consistent with the notion that the *Setdb1* gene plays a central role in H3K9 trimethylation, its disruption led to a profound decline in the level of H3K9me3 on all MGA-bound genomic sites, regardless of the cluster to which they belong (Figure S5). Our evaluation also revealed that only cluster 1 and 3 genes, but not genes categorized into other clusters, showed apparent decline in the levels of H3K9me3 modification due to *Atf7ip* gene disruption (Figure 2A, a broader view is provided in Figure S6). To unbiasedly analyze these differences, we treated these data statistically. These analyses confirmed the significant decline in the modification levels by *Atf7ip* gene disruption with cluster 3 genes. However, rather weak significance was suggested with cluster 5 and 7 genes. However, no such statistically significant difference was found with cluster 1 genes (Figure 2B). Our analyses with *Zmym2*-null ESCs (Figure S7) revealed that none of the gene clusters showed a statistically significant decline in the H3K9me3 levels due to *Zmym2* gene disruption (Figures S8A and S8B), indicating that none of the gene clusters markedly depends on ZMYM2 for the acquisition of H3K9me3 modification. Consistent with this, ChIP-qPCR analyses of histone modification data revealed that the promoters of *Mael* and *Stk31* genes, both of which are representative meiosis-related genes classified into cluster 3, did not show notable reduction of any repressive histone marks, including H3K9me3 modification, due to disruption of the *Zmym2* gene (Figure S8C). Meanwhile, the levels of H3K9me2 and H3K9me3, but not those of H2AK119ub and H3K27me3, were significantly reduced in these promoters by *Atf7ip* gene disruption (Figure 2C). However, levels of H3K9me2 and H3K9me3 did not markedly decrease to the background level, indicating that other unknown mechanisms operate in parallel with the ATF7IP-dependent mechanism or specifically in ATF7IP-null conditions. To further confirm the decline in the level of H3K9me3 because of *Atf7ip* gene disruption in ESCs, we forcibly expressed ATF7IP in *Atf7ip*-null ESCs using the *piggy*Bac system^35^ and confirmed the significant elevation in the H3K9me3 levels on promoters of *Meiosin*, *Mael*, and *Stk31* genes due to ATF7IP overexpression (Figure S9). Although KAP1 functions as a bridging factor between SETDB1 and a protein belonging to the KRAB-ZFP superfamily, such as ZFP809 that binds to ERVs directly to recruit the SETDB1 complex to ERVs,^32^^,^^36^ any other factors should be able to substitute for the function of KAP1/KRAB-ZFP of recruiting SETDB1 on MGA-bound genomic sites because KAP1 did not show appreciable levels of binding on any clusters. In addition to the Setdb1-binding domain, ATF7IP bears an additional functional domain termed the FNIII domain, whose functional significance remains elusive (Figure 2D). ZMYM2 and MGA are the top-ranked proteins that have been identified as factors able to interact with this domain.^34^ Furthermore, MGA has an intrinsic ability to bind directly to the genome.^24^ Because of these characteristics, we hypothesized that MGA may substitute for the functions of both KAP1 and KRAB-ZFP, at least on cluster 3 genes whose H3K9me3 mark is dependent on ATF7IP, but not ZMYM2. Namely, MGA may recruit SETDB1 onto meiosis-related genes via its interaction with ATF7IP. As an initial test to confirm this hypothesis, we compared the levels of binding between wild-type ATF7IP and its mutant lacking the FNIII domain on cluster 3 genes in ESCs using ChIP-sequence data deposited by Tsusaka et al.^34^ (Figure 2E). These analyses revealed that removal of the FNIII domain from ATF7IP was accompanied by a substantial decline in the levels of binding to cluster 3 genes. Consistent with this, browser snapshots of *Mael* and *Stk31* genes showed less efficient binding of the ATF7IP mutant compared with that of its wild-type counterpart on both gene promoters (Figure 2F). To further substantiate the above hypothesis, we newly generated mutant ESCs expressing ATF7IP that lacks the FNIII domain using the CRISPR–Cas9 system (Figures S10A–S10C) and then examined the effect of this mutation on H3K9me3 levels on cluster 3 genes. These analyses revealed that, like the complete loss of ATF7IP (Figures 2A and 2B), the loss of only its FNIII domain also led to a significant decline in the level of H3K9me3 modification of cluster 3 genes (Figure 2G).

### MGA binds to ATF7IP via its FAM domain

**Figure 3:**
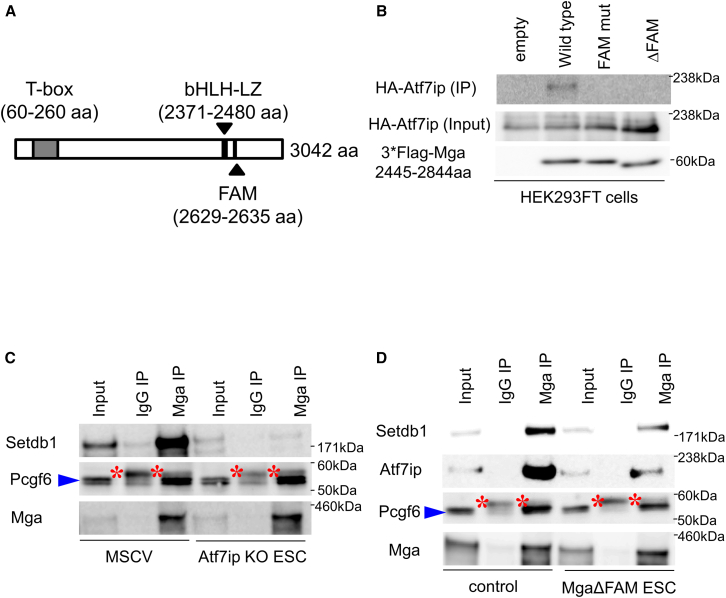
Biochemical characterization of interaction between ATF7IP and MGA (A) Schema of functional domains of MGA. (B) Coimmunoprecipitation analyses of HA-tagged ATF7IP with wild-type and mutant MGAs. Expression vectors for the Flag-tagged carboxy-terminally located portion of MGA with the wild-type sequence and its mutants [in which the portion of cDNA corresponding to FAM is either deleted or mutated to convert the amino acid sequence of FAM (IVNVTSLAA) to ***R***VN***R***TS***R***AA] were individually introduced into HEK293FT cells together with the expression vector for HA-tagged ATF7IP. Then, coimmunoprecipitation analyses were conducted using whole-cell extracts prepared 48 h after transfection. (C) Coimmunoprecipitation analyses of MGA with SETDB1 in parental and *Atf7ip*-null ESCs. In the analysis, interaction between PCGF6 and MGA was also examined as a reference. Blue arrowhead and red asterisk indicate signals from PCGF6 protein and heavy chain of immunoglobulin, respectively. (D) Coimmunoprecipitation analyses of MGA with SETDB1 in parental and MGAΔFAM ESCs. The interaction between PCGF6 and MGA was also examined as a reference as in (C). Blue arrowhead and red asterisk are used as in (C).

Most ATF7IP-interacting proteins, including MGA, possess a specific amino acid sequence termed FAM, FNIII domain of ATF7IP-interacting motif^34^ (Figure 3A). Therefore, we next examined whether the FAM sequence of MGA serves as a direct binding site for ATF7IP. Our coimmunoprecipitation analyses revealed that 3×Flag-tagged carboxy-terminal domain of MGA overexpressed in HEK293FT cells bound to HA-tagged ATF7IP, which was also efficiently exogenously expressed in the cells, but mutation of FAM (IVNVTSLAA to ***R***VN***R***TS***R***AA) or its deletion resulted in loss of its interaction with ATF7IP (Figure 3B). Furthermore, our analyses revealed that MGA failed to interact with SETDB1 in the ATF7IP-null background in ESCs, although MGA retained its ability to bind to PCGF6 efficiently (Figure 3C). These results indicate that, like the originally identified FAM in ZMYM2,^34^ FAM identified within MGA did function as the binding site for ATF7IP bound to SETDB1. To further examine the importance of MGA’s FAM for the interaction with ATF7IP and SETDB1 in ESCs, we generated ESCs expressing an MGA internal deletion mutant that lacks the FAM domain again using the CRISPR–Cas9 system (Figures S11A and S11B). After confirming that removal of the FAM domain from MGA affected neither its protein stability nor the total amounts of repressive histone modification marks (Figures S11C and S11D), we conducted coimmunoprecipitation analyses using the genetically engineered ESCs. In accordance with our hypothesis, the amounts of SETDB1 and ATF7IP that coimmunoprecipitated with MGA, but not those of PCGF6, were substantially decreased due to the mutation of MGA, although deletion of the FAM domain did not completely disrupt their interaction with MGA (Figure 3D). The fact that ATF7IP retained substantial ability to bind to the MGA mutant (MGAΔFAM) may indicate that ATF7IP can also bind to MGA through a domain other than FAM or that ATF7IP binds to MGA indirectly via its interaction with another component of PRC1.6. We also examined the consequence of *Zmym2* gene disruption on the interaction of MGA with ATF7IP and SETDB1. In agreement with the finding of *Zmym2* gene disruption having no notable effect on H3K9me3 levels at any of the gene clusters (Figures S8A and S8B), our coimmunoprecipitation analyses revealed that MGA retained its full capacity to interact with SETDB1 and ATF7IP in the absence of ZMYM2 (Figure S12).

### Crucial role of FAM domain of MGA for the repression of meiosis-related gene expression

**Figure 4:**
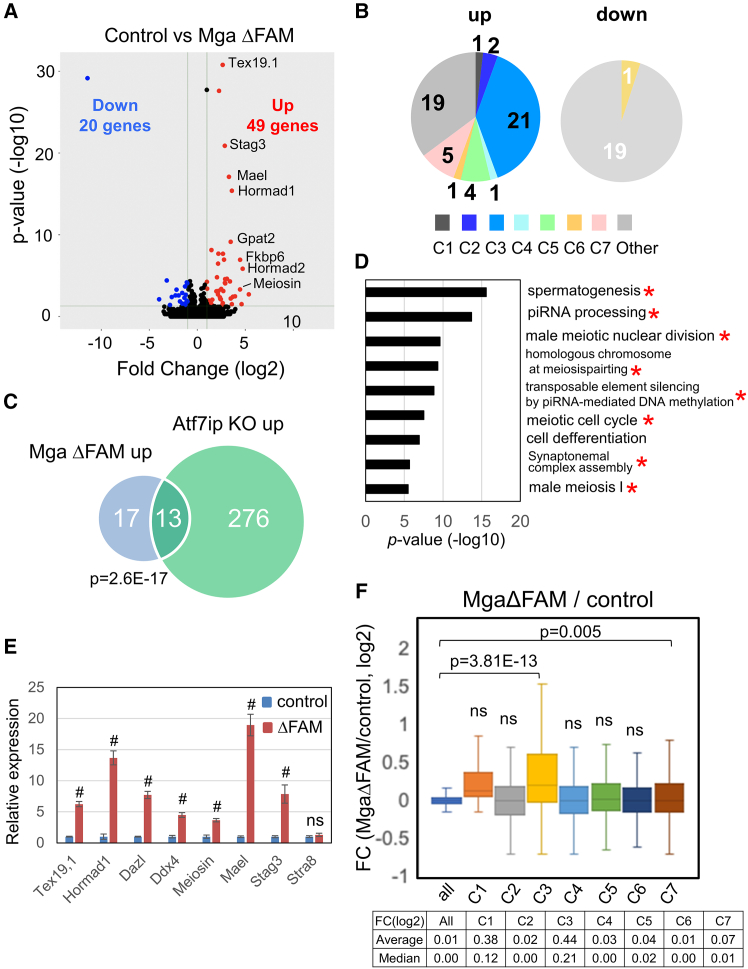
Deletion of FAM domain from MGA was accompanied by the upregulation of meiosis-related genes in ESCs (A) Volcano plot presenting genes differentially expressed between parental ESCs and those producing MGA lacking the FAM domain. Representative meiosis-related genes are indicated by a red dot with a gene symbol. Lists of genes up- or downregulated more than 2-fold due to the removal of FAM from MGA are provided in Table S3A with the information of the classified cluster number. (B) Skewed distribution of activated genes, but not those repressed, in MGAΔFAM ESCs into the cluster 3 gene group. Forty-nine activated genes in MGAΔFAM ESCs were subdivided according to seven clusters identified in Figure 1A and the information is provided as a pie chart (left panel). The same procedure was conducted for 20 downregulated genes, data for which are shown in the right panel. (C) Venn diagram showing prominent overlap of activated genes between *Atf7ip*-null ESCs and those producing MGA mutant (ΔFAM). Among 49 activated genes in the MGA mutant ESCs, 19 genes that were not classified into any gene cluster were removed from the gene list and the remaining 30 genes were used to examine the extent of overlap with genes activated by *Atf7ip* gene disruption. *p* value for the significance of the overlap between the two gene sets was calculated by a hypergeometric test. (D) GO analyses for assessing the effect of removal of the FAM domain from MGA in ESCs. GO analyses were conducted using 30 genes selected in C. GO terms whose *p* value is lower than 1 × 10^−5^ are presented. Red asterisks indicate the terms related to meiosis/gametogenesis. (E) qPCR analyses of the expression of eight representative meiosis-related genes that are repressed directly by PRC1.6, except for *Stra8* in wild-type and MGAΔFAM ESCs. Data represent mean ± SD of three independent experiments. Two-tailed unpaired Welch’s t test was conducted to examine statistical significance. #, *p* < 0.05; ns, *p* > 0.05. (F) Quantitative analysis of the changes in expression levels of all genes and seven gene clusters due to the removal of FAM from MGA using a boxplot. Data for transcriptional change from each gene cluster were compared with those from all annotated coding and noncoding genes (*n* = 55,492). Statistical significance was determined using one-way ANOVA followed by Bonferroni’s post hoc test. ns, *p* > 0.05.

To unbiasedly explore the functional relevance of the FAM domain of MGA, we compared global expression profiles between ESCs expressing MGAΔFAM mutant and their control counterparts by RNA sequence analyses (Figure 4A). We found that 49 and 20 genes were significantly up- and downregulated due to the loss of the FAM domain from MGA. First, we investigated whether these genes were included in any of the seven clusters of MGA-bound genes (Figure 4B; Table S3A). Almost all downregulated genes (19 out of 20) and some upregulated ones (19 out of 49) were not classified into any of the clusters shown in Figure 1A, indicating that MGA does not bind to these genes. It is thus conceivable that the alteration in expression levels of these MGA-unbound genes due to loss of the FAM domain of MGA does not represent the direct consequence of MGA mutation, but rather represents noise and/or is at least an indirect consequence. However, because 35 genomic sites covering 30 out of 49 upregulated genes were included in one of the seven clusters, with enrichment in cluster 3 being predominant, we explored the characteristics of these 30 genes. First, our analyses revealed that PCGF6 was highly accumulated on these 30 genes when compared with the level on all MGA-bound genes (Figure S13A), consistent with the fact that many of them are genes categorized into cluster 3. Our data also showed relatively high accumulation of ATF7IP, SETDB1, and H3K9me3 on these 30 genes as well (Figures S13B–S13D), indicating that at least upregulation of these 30 genes represents a direct consequence of removal of the FAM domain from MGA. In accordance with our model, these 30 genes identified with the MGA mutant ESCs significantly overlapped with those upregulated in A*tf7ipKO* ESCs (Figure 4C). GO analyses to assess the overall molecular functions of these 30 activated genes revealed the significant enrichment of genes related to meiosis (Figure 4D; Table S3B). Next, we conducted qPCR on eight meiosis-related genes and found that seven of them classified into cluster 3, with the exception of unclassified *Stra8*, exhibited significant elevation in their expression levels due to removal of the FAM domain from MGA, confirming the RNA-seq and qPCR data (Figure 4E). Next, we individually compared the mean changes in expression levels of seven gene clusters resulting from removal of the FAM domain of MGA with that of all annotated coding and noncoding genes (*n* = 55,492) in ESCs (Figure 4F). These analyses revealed that only cluster 3 and 7 genes, but not those in other gene clusters, exhibited significant increases in their mean expression levels, with the increase of cluster 3 genes being much more significant than that of cluster 7 genes. This is consistent with the idea that many genes classified into cluster 3 are directly subjected to MGA/ATF7IP/SETDB1 complex-dependent repression. However, unexpectedly, we found that most cluster 3 genes did not show such activation by the *Atf7ip* gene disruption in ESCs (Figure S14). We discuss this apparent discrepancy later (see discussion).

### FAM domain of MGA-dependent deposition of H3K9me3 mark on meiosis-related genes

**Figure 5:**
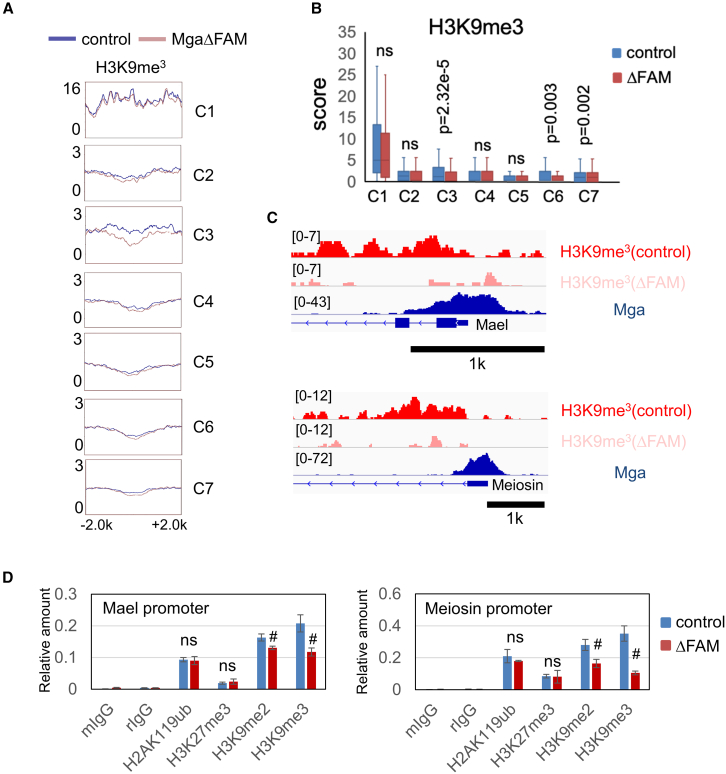
MGA-binding cluster 3 genomic regions depend on FAM domain of MGA for their H3K9me3 (A) Comparison of the effect of removal of the FAM domain from MGA on H3K9me3 levels among seven MGA binding site-containing gene cluster sets in ESCs. An image covering a broader region (−10k to +10k) is provided in Figure S15. (B) Quantitative analysis of the difference in H3K9me3 levels due to the removal of FAM from MGA in ESCs in each gene cluster using a boxplot. Statistical significance was assessed using two-tailed unpaired Welch’s t test. ns, *p* > 0.05. (C) Genomic snapshots showing the densities of H3K9me3 marks over *Mael* and *Meiosin* genes in wild-type ESCs and those producing MGA lacking the FAM domain. Data of MGA density on these genes are provided as a reference. (D) ChIP-qPCR analyses of H2AK119ub, H3K27me3, H3K9me2, and H3K9me3 on *Mael* and *Meiosin* gene promoters using wild-type and MGAΔFAM ESCs. Data represent mean ± SD of three independent experiments. Two-tailed unpaired Welch’s t test was conducted to examine statistical significance. #, *p* < 0.05; ns, *p* > 0.05.

We next examined the H3K9me3 modification levels of ESCs expressing the ΔFAM mutant of MGA. Metagene plots indicated that genes classified into cluster 3 showed apparent reductions in their H3K9me3 modification levels due to removal of the FAM domain from MGA in ESCs, while genes classified into other clusters including cluster 1 did not show noticeable alterations in their levels (Figure 5A, a broader view is provided in Figure S15). As in Figure 2B, we treated these data statistically and again confirmed the significant effect of removal of the FAM domain from MGA rather specifically on cluster 3 genes, although somewhat weak links between the FAM domain and H3K9me3 were evident with clusters 6 and 7 (Figure 5B). Figure 5C shows representative examples of cluster 3 genes (*Mael* and *Meiosin*) that exhibited substantial reductions in the levels of this histone modification due to loss of the FAM domain from MGA. Our ChIP-qPCR analyses verified that di- and trimethylation levels of H3K9 declined significantly due to removal of the FAM domain from MGA on these two gene promoters in ESCs, while this MGA mutation did not affect the level of H2AK119ub or H3K27me3 (Figure 5D). Furthermore, we examined whether decline in the level of H3K9me3 because of the removal of FAM from MGA in ESCs could be counteracted by the overexpression of wild-type MGA in MGA-mutant ESCs. As expected, our data demonstrated the significant elevation in the H3K9me3 levels on promoters of *Meiosin* and *Mael* genes due to MGA overexpression (Figure S16).

**Figure 6:**
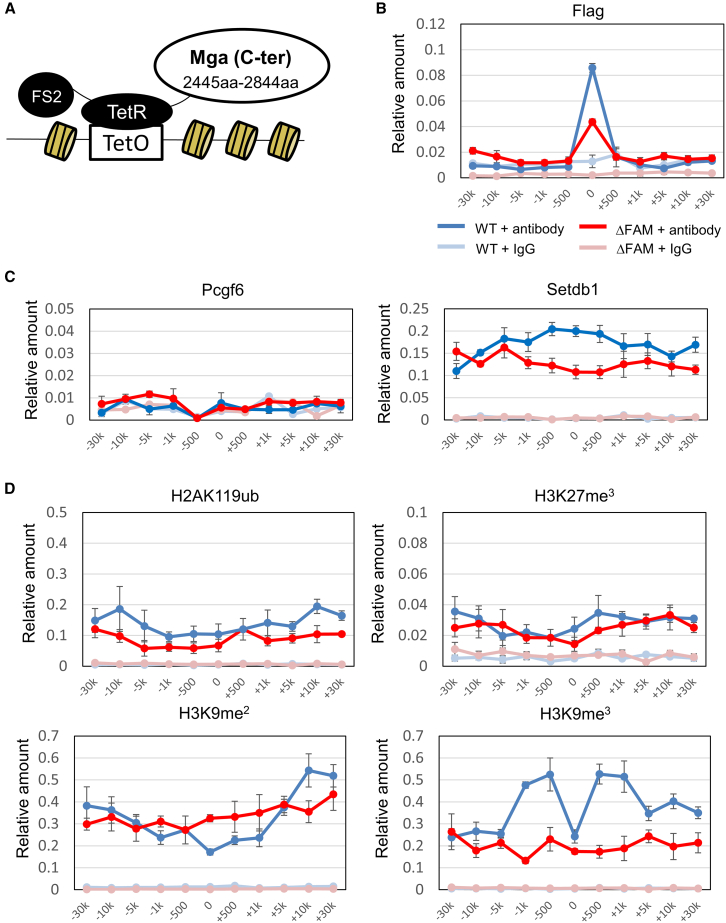
Demonstration of FAM domain of MGA-dependent deposition of H3K9me mark by TetR/TetO-based *de novo* targeting assay (A) Schematic illustration of recruitment of the carboxy-terminal portion of MGA that is fused to TetR on TetO array in ESCs. In the analyses, the carboxy-terminal portion of MGA with the wild-type amino acid sequence (2,445–2,844) and that with no FAM sequence were used. Mouse ESCs used in the analyses possess a TetO array that is flanked by BAC-derived human genomic DNA in chromosome 7^37^. (B) ChIP-qPCR analyses of Flag-tagged TetR fused to MGA with the wild-type or mutant amino acid sequence. (C) ChIP-qPCR for comparing the levels of recruitment of PCGF6 and SETDB1 on and around the TetO array between ESCs expressing Flag-tagged TetR/MGA fusion protein with FAM domain and those bearing the fusion protein with no FAM domain. (D) ChIP-qPCR for comparing the levels of H2AK119ub, H3K27me, H3K9me2, and H3K9me3 marks on and around the TetO array between the same set of ESCs used in (C).

To obtain independent lines of evidence of the involvement of the FAM domain of MGA in the H3K9me3 modification, we used a synthetic system developed by Blackledge et al.^37^ (Figure 6A). In this system, any protein of interest is exogenously expressed as a fusion protein with Tet repressor DNA binding domain (TetR) in mouse ESCs, whose chromosome 8 carries bacterial artificial chromosome (BAC)-derived human genomic DNA with Tet repressor DNA binding sites (TetO). First, we confirmed by ChIP-qPCR that TetR proteins fused to either the carboxy-terminal portion of MGA with the wild-type amino acid sequence or its mutant lacking the FAM domain bound efficiently to TetO sites (Figure 6B). Because only a small portion of MGA containing the FAM domain was used as bait, the PRC1.6 complex was not expected to be constructed on a TetO site, but the SETDB1/ATF7IP complex could be expected to be recruited via the interaction between the FAM domain of MGA and the FNIII domain of ATF7IP. As expected, we confirmed that SETDB1, but not PCGF6, was recruited to the TetO site and its flanking region in a functional FAM domain-dependent manner (Figure 6C). Then, we applied ChIP-qPCR analyses to examine whether newly incorporated histone modifications related to transcriptional repression differed in their levels in relation to the presence or absence of the FAM domain of MGA (Figure 6D). We found that, while the levels of H2AK119ub and H3K27me3 were comparable between these two fusion proteins, forced production of TetR fusion protein with the wild-type MGA amino acid sequence, but not that fused to the corresponding portion of MGA lacking the FAM domain, resulted in significant elevation in the level of H3K9me3 modification, further reinforcing the notion that MGA is directly involved in recruiting ATF7IP/SETDB1 complex via its FAM domain for deposition of the H3K9me3 modification. We considered that the lower H3K9me3 levels at the center of TetO sites than at other regions in the vicinity were due to TetR proteins that occupied the surface of TetO sites. Interestingly, we also found that the level of H3K9me2 modification on the TetO site and in its vicinity declined slightly due to the forced production of TetR fusion protein with the wild-type MGA amino acid sequence. We attributed this decline to the use of this modification as a substrate for the production of H3K9me3 modification. We cannot completely rule out the possibility that differences in the levels of recruitment of SETDB1 and deposition of H3K9me3 modification between TetR with the wild-type MGA sequence and that with the mutant sequence were attributable to differences in the efficiency of their binding to TetO sites. However, this is considered unlikely because TetR with the wild-type MGA sequence showed these activities rather specifically in the vicinity of TetO sites, whereas the mutant did not show even marginal activity across rather broader regions for both events, reinforcing our conclusion that these differences represent intrinsic functional differences between TetRs with wild-type and mutant sequences of MGA.

## Discussion

Among six distinct PRC1 subtypes, we and others previously reported that PRC1.6 plays crucial roles in establishing a transcriptionally repressed state on meiosis-related genes to prevent their ectopic expression in ESCs.^17^^,^^18^^,^^19^ However, many meiosis-related genes, such as *Mael* and *Meiosin* bear substantial numbers of repressive H3K9me3 histone marks, as well as H2K119ub and H3K27me3 histone modifications.^11^ Because the acquisition of H3K9me3 is a prerequisite for robust repression by recruiting HP1 and DNA methyltransferase,^38^^,^^39^^,^^40^^,^^41^ the chromatin state on meiosis-related genes in ESCs is regarded as the state at least one step further along toward the establishment of a robustly repressed state.

Similar to meiosis-related genes, ERVs are marked substantially with H3K9me3 modification in ESCs, in which SETDB1 plays a central role in depositing H3K9me3 on ERVs while its nuclear localization is dependent on its binding partner, ATF7IP.^29^^,^^30^^,^^31^^,^^33^^,^^34^ In addition to Setdb1 binding domain, ATF7IP possesses an additional functional domain termed the fibronectin (FNIII) domain.^29^^,^^30^ However, the functional importance of this domain has remained unclear because ERVs that exhibited elevation in their expression levels due to *Atf7ip* gene disruption have been shown to regain their repressed state not only by the forced production of wild-type ATF7IP but also by the ATF7IP mutant lacking its FNIII domain.^34^ However, that study also showed that many genes related to meiosis were exceptional because their elevated expression in *Atf7ip*-null ESCs was repressed only by the overexpression of wild-type ATF7IP, but not by the ATF7IP mutant. One of the most important outcomes of the present study is that it uncovered why the FNIII domain of ATF7IP is dispensable and essential for the repression of ERVs and meiosis-related genes, respectively. Namely, we demonstrated that the difference in dependence on the FNIII domain of ATF7IP for the transcriptional repression between ERVs and meiosis-related genes reflects their difference in the manner of recruitment of the ATF7IP/SETDB1 complex. Specifically, KAP1 plays crucial roles for the targeting of the complex to ERVs, with the aid of KRAB-ZFP superfamily proteins such as ZFP809.^29^^,^^30^ By contrast, KAP1 is not involved in the targeting of the complex to meiosis-related genes, but instead the FNIII domain of ATF7IP is used for recruitment to meiosis-related genes via its interaction with the FAM domain of MGA. Consistent with this, our data demonstrated that loss of FAM from MGA was accompanied by rather specific activation of cluster 3 genes, among which meiosis-related genes are particularly prominent. However, we also encountered an apparently discrepant result, namely, that no such obvious activation of these genes was observed with *Atf7ip*-null ESCs. Recently, a new role of the SETDB1/ATF7IP complex was uncovered.^42^ In this noncanonical role, the complex contributes to the construction of a unique topological structure enriched with activating histone marks such as H3K4me3 and H3K27Ac in the genome by functioning together with COHESIN, in an H3K9me3-independent manner. We assumed that the different effects on cluster 3 gene expression between the removal of FAM from MGA and the loss of ATF7IP were due to their different effects on the noncanonical activity of SETDB1, with the MGA mutation being supposed to affect only the canonical activity of SETDB1, whereas *Atf7ip* gene knockout would affect both canonical and noncanonical activities of SETDB1. Namely, it can be assumed that the disruption of this noncanonical activity of STEDB1 by the complete loss of ATF7IP masks the apparent changes due to impairment of the canonical activity of SETDB1, as, unlike the canonical activity of SETDB1, its noncanonical activity is often, if not always, associated with positive regulation.^42^

**Figure 7:**
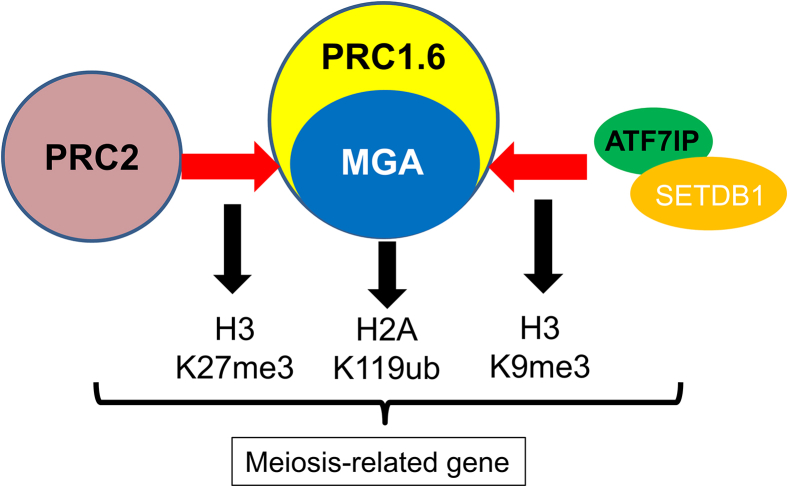
Model illustrating MGA FAM domain-dependent recruitment of SETDB1/ATF7IP for deposition of H3K9me3 mark on meiosis-related genes In this model, MGA exerts a dual role for establishing a transcriptionally repressive state of meiosis-related genes in ESCs: first contributing to the establishment of a PRC1/PRC2-dependent repressive state by functioning as a scaffolding component of PRC1.6 and then generating an epigenetic state that acts as a foundation for a more robustly repressed state by depositing H3K9me3 modification via its interaction with STEDB1/ATF7IP complex.

Taking the obtained findings together, the present study demonstrates that MGA, which plays central roles in the establishment of a transcriptionally repressed state with the histone modification marks H2K119ub and H3K27me3 as a scaffolding component of PRC1.6,^17^^,^^18^^,^^19^ also contributes to the deposition of histone H3K9me3 marks by providing a binding site for the ATF7IP/SETDB1 complex, as depicted in Figure 7. Although the acquisition of histone H3K9me3 modification is generally known to be a key step that leads to the establishment of a robustly repressed state with DNA methylation,^38^^,^^39^^,^^40^ it is not known whether this MGA-dependent histone H3K9me3 deposition indeed leads to DNA methylation of CpG-enriched promoters of meiosis-related genes for the acquisition of an even more robustly repressed state. We hope that the MGA-centered mechanism of transcriptional repression of meiosis-related genes in ESCs that we uncovered in this study provides a robust foundation from which to address this issue.

### Limitations of the study

In this study, we have demonstrated in ESCs that MGA, a scaffolding component of PRC1.6, one of the subtypes of PRC1, recruits the ATF7IP/SETDB1 complex that deposits the H3K9me3 epigenetic modification on a subset of genes, particularly cluster 3 genes, many of which are associated with meiosis. This in turn establishes a more robustly repressed state in these cells. However, we do not know whether the same mechanism operates in early embryonic cells such as the inner cell mass of the blastocyst *in vivo*. Another limitation of our study is that we cannot completely rule out the possibility that this mechanism does not occur in other species such as humans, but operates exclusively in mouse cells, because we elucidated the mechanism using mouse ESCs. Finally, we do not know whether MGA-dependent acquisition of trimethylation of lysine 9 of histone H3 leads to eventual methylation on DNA for an even more robustly repressed state.

## Resource availability

### Lead contact

Further information and requests for resources and reagents should be directed to and will be fulfilled by the lead contact, Akihiko Okuda (akiokuda@saitama-med.ac.jp).

### Materials availability

This study did not generate new unique reagents.

### Data and code availability

• Data: The RNA-seq and ChIP-seq datasets generated in this study are available at Gene Expression Omnibus (GEO) under accession number GSE268907.
• Code: All data were analyzed using publicly available packages as described in detail in method details of STAR Methods.
• Other items: Any additional information required to reanalyze the data reported in this article will be shared by the lead contact upon reasonable request.

## Acknowledgments

We are indebted to Dr. Yoichi Shinkai for the provision of *Atf7ip*-KO ESCs and helpful discussions. We also thank Edanz (https://jp.edanz.com/ac) for editing a draft of this manuscript. This work was supported in part by the 10.13039/501100001700Ministry of Education, Culture, Sports, Science and Technology (MEXT), Japan. K.U. and A.O. are recipients of grants from the 10.13039/501100001691Japan Society for the Promotion of Science (JSPS) KAKENHI with grant numbers 23K06391 and 23K27369, respectively. K.U. and A.S. are recipients of grants from 10.13039/100007449Takeda Science Foundation as research representatives. J.R.K. is supported by the 10.13039/100010269Wellcome Trust (209400/Z/17/Z).

## Author contributions

Conceptualization, K.U., A.O., and A.S.; methodology, K.U.; formal analysis, K.U. and M.H.; investigation, K.U., M.N., and A.S.; data curation, K.U. and M.H.; writing – original draft, K.U.; writing – review & editing, R.J.K., A.O., and A.S.; funding acquisition, K.U., A.O., and A.S.; resources, R.J.K.; supervision, A.O. and A.S.

## Declaration of interests

The authors declare no competing interests.

## STAR★Methods

### Key resources table

**Table:** 

REAGENT or RESOURCE	SOURCE	IDENTIFIER
**Antibodies**		

Anti-histone H3 antibody	Cell Signaling Technology	Cat# 9715S; RRID: AB_331563
Anti-histone H3K27me3 antibody	Millipore	Cat# 07–499; RRID: AB_310624
Anti-histone H3K9me2 antibody	abcam	Cat# ab1220; RRID: 449854
Anti-histone H3K9me3 antibody	abcam	Cat# ab8898; RRID: 306848
Anti-histone H2A antibody	Cell Signaling Technology	Cat# 2578S; RRID: AB_2118804
Anti-histone H2AK119ub antibody	Cell Signaling Technology	Cat# 8240S; RRID: AB_10891618
Anti-LAMIN A/C antibody	Santa Cruz Biotechnology	Cat# sc-20681; RRID: AB_648154
Anti-MGA antibody	abcam	Cat# ab214814
Anti-ATF7IP antibody	abcam	Cat# ab84497; RRID: AB_1861009
Anti-SETDB1 antibody	Proteintech	Cat# 11231-AP; RRID: AB_2186069
Anti-ZMYM2 antibody	abcam	Cat# ab106624; RRID: AB_10863065
Anti-PCGF6 antibody	abcam	Cat# ab200038
Anti-βactin antibody	Santa Cruz Biotechnology	Cat# sc-47778; RRID: AB_626632
Anti-HA-tag antibody	abcam	Cat# 3724S; RRID: AB_1549585
Anti-Flag M2 antibody	Sigma-Aldrich	Cat# F3165; RRID: AB_259529
Normal mouse IgG	Cell Signaling Technology	Cat# 5415S; RRID: AB_10829607
Normal rabbit IgG	Cell Signaling Technology	Cat# 2729S; RRID: AB_1031062
Goat anti-mouse IgG HRP-linked antibody	Cell Signaling Technology	Cat# 7076
Goat anti-rabbit IgG HRP-linked antibody	Cell Signaling Technology	Cat# 7074
Mouse TrueBlot ULTRA: Anti-Mouse IgG HRP	ROCKLAND	Cat# 18-8817-31
Rabbit TrueBlot ULTRA: Anti-Rabbit IgG HRP	ROCKLAND	Cat# 18-8816-31

**Chemicals, peptides, and recombinant proteins**		

Lipofectamine 2000	Thermo Fisher Scientific	Cat# 11668027
Puromycin	MERCK	Cat# P8833
polybrene	Nacalai Tesque, Japan	Cat# 17736-44
SYBR Green qPCR Master Mix	Thermo Fisher Scientific	Cat# A66732
TaqMan Fast Universal Master Mix	Thermo Fisher Scientific	Cat# 4352042
Magna ChIP Protein A Magnetic Beads	MERCK	Cat# 16-661

**Critical commercial assays**		

Illumina Stranded Total RNA Pre, Ligation with Ribo-Zero Plus	Illumina	20040529

**Deposited data**		

RNA-seq data for MgaDFAM ESCs and their control and ChIP-seq (histone H3K9m3) data for MgaDFAM and ATF7IPDFNIII ESCs and their respective control ESCs	This paper	GEO accession: GSE268907
ChIP-seq data of mouse ESCs for MGA and PCGF6	Stielow et al.^19^	PRJEB66757
ChIP-seq data of Atf7ip-null ESCs with Flag-tagged wildtype or DFNIII mutant of ATF7IP	Tsusaka et al.^34^	PRJNA664286
ChIP-seq data of mouse ESCs for ZMYM2	Yang et al.^43^	GEO accession: GSE119818
ChIP-seq data of mouse ESCs for SETDB1	Warrier et al.^42^	GEO accession: GSE123244
ChIP-seq data of mouse ESCs for KAP1	Rowe et al.^44^	GEO accession: GSE41903
ChIP-seq data of control mouse ESCs and Atf7ip-null ESCs for histone H3K9me3	Butz et al.^45^	GEO accession: GSE176461
ChIP-seq data of control mouse ESCs and Setdb1-null ESCs for histone H3K9me3	Barral et al.^46^	GEO accession: GSE171749
ChIP-seq data of control mouse ESCs and Zmym2-null ESCs for histone H3K9me3	Graham- Paquin et al.^47^	GEO accession: GSE214197

**Experimental models: Cell lines**		

EBRTcH3 ESCs	Masui et al.^48^	N/A
MSCV-GFP ESCs	Fukuda et al.^49^	N/A
Atf7ip-KO ESCs	Tsusaka et al.^50^	N/A
Setdb1-KO ESCs	Barral et al.^46^	N/A
Zmym2-KO ESCs	This paper	N/A
E14TetIN ESCs	Blackledge et al.^37^	N/A
Mga-ΔFAM ESCs	This paper	N/A
HEK293FT	Laboratory stock	N/A

**Oligonucleotides**		

Synthetic oligonucleotides used in this study were listed in Table S1	This study	N/A
*Tex19.1*	Thermo Fisher Scientific	Mm93953368_s1
*Hormad1*	Thermo Fisher Scientific	Mm00471448_m1
*Dazl*	Thermo Fisher Scientific	Mm03053726_s1
*Ddx4*	Thermo Fisher Scientific	Mm00802445_m1
*Meiosin*	Thermo Fisher Scientific	Mm01305445_m1
*Mael*	Thermo Fisher Scientific	Mm01293195_m1
*Stra8*	Thermo Fisher Scientific	Mm00486573_m1
*Gapdh*	Thermo Fisher Scientific	Mm99999915_g1

**Recombinant DNA**		

Plasmid: pLentiCRISPRv2	Sanjana et al.^51^	Addgene Plasmid #52961
Plasmid: psPAX2	One of addgene plasmids deposited by Dr. Didier Trono in Switzerland	Addgene Plasmid #12260
Plasmid: pLP/VSVG	NovoPro Bioscience	Cat# V010445
Plasmid: pCAG/HA-Atf7ip	This study	N/A
Plasmid: pCAG/HA-Mga	This study	N/A
Plasmid: pCAG/3xFlag-Mga C-ter (aa2445-2844)	This study	N/A
Plasmid: pCAG/3xFlag-Mga C-ter FAM mut	This study	N/A
Plasmid: pCAG/3xFlag-Mga C-ter ΔFAM	This study	N/A
Plasmid: pCAG/3xFlag-Mga, PGK-puro	This study	N/A
Plasmid: pCAGFS2TetR	Blackledge et al.^37^	N/A
Plasmid: pCAGFS2TetR/Mga C-ter	This study	N/A
Plasmid: pCAGFS2TetR/Mga C-ter ΔFAM	This study	N/A
Plasmid: pPB-CAG/Atf7ip	This study	N/A
Plasmid: *piggyBac* transposase expression vector	Yusa et al.^35^	N/A

**Software and algorithms**		

R version 4.1.0	R Development Core Team	https://www.r-project.org
EdgeR version 3.34.0	Robinson et al.^52^	https://bioconductor.org/about/
Sickle version 1.33	Joshi NA and Fass JN	https://github.com/najoshi/sickle
Bowtie2 version 2.4.1	Langmead and Salzberg^53^	https://bowtie-bio.sourceforge.net/bowtie2/index.shtml
Samtools version 1.10	Li et al.^54^	http://htslib.org; RRID: SCR_002105
MACS2 version 2.2.7.1	Zhang et al.^55^	https://hbctraining.github.io/Intro-to-ChIPseq/lessons/05_peak_calling_macs.html
GREAT version 4.0.4	McLean et al.^56^	http://great.stanford.edu/public/html
deepTools version 3.4.3	Ramirez et al.^57^	https://github.com/deeptools/deepTools
HISAT2 version 2.1.0	Kim et al.^58^	http://deahwankimlab.gifhub.io/hisat2 RRID: SCR_015530
StringTie version 2.1.2	Pertea et al.^59^	https://ccb.jhu.edu/software/stringtie/
dplyr version 1.0.10	Wickham et al.^60^	https://dplyr.tidyverse.org
ggplot2 version 3.3.5	Wickham^61^	https://ggplot2.tidyverse.org

### Experimental model and study participant details

#### Cell lines and cell culture

MSCV-GFP mouse ESCs including their *Atf7ip* gene-knockout derivatives^49^^,^^50^ and EBRTcH3 mouse ESCs^48^ were kindly provided by Professor Yoichi Shinkai at RIKEN Cluster for Pioneering Research in Japan and Professor Hitoshi Niwa at Kumamoto University, Japan, respectively, while E14IN mouse ESCs are from laboratory stocks of Robert J. Klose.^37^ Mga-ΔFAM and Zmym2-null ESCs were generated using the CRISPR/Cas9 system with EBRTcH3 ESCs as parental cells, as detailed in the section “Genetic manipulation in ESCs by lentivirus-mediated CRISPR–Cas9 system” of method details. MSCV-GFP mouse ESCs and their derivatives were cultured in Dulbecco’s modified Eagle’s medium (DMEM) supplemented with 15% Knockout Serum Replacement, 1% fetal bovine serum (FBS), 0.1 mM 2-mercaptoethanol (2-ME), leukemia inhibitory factor (LIF) (1000 units/mL), and 1× nonessential amino acids (NEAA), while other ESC lines were cultured in Glasgow minimal essential medium supplemented with 10% FBS, 0.1 mM 2-ME, LIF (1000 units/mL), 1× NEAA, and 1× sodium pyruvate.

### Method details

#### Genetic manipulation of ESCs by lentivirus-mediated CRISPR–Cas9 system

*Mga*, *Atf7ip*, and *Zmym2* genes were individually edited at exons 24, 15, and 2, respectively, using a lentivirus-mediated CRISPR–Cas9 system. pLentiCRISPRv2 (Addgene #52961) carrying specific oligonucleotides listed in Table S1 for editing the *Mga*, *Atf7ip*, or *Zmym2* gene was introduced into HEK293FT cells together with psPAX2 (Addgene #12260) and pLP-VSVG (Invitrogen) vectors by lipofection. Lentiviruses recovered from transfected cells were used to infect parental ESCs using polybrene (8 μg/mL). Then, the infected ESCs were subjected to puromycin selection (1 μg/mL) for 6 days. The resultant puromycin-resistant ESC colonies were subsequently recovered individually and their genomic DNA was used to identify ESC clones whose genomes had been successfully modified as expected.

#### Forced expression of wild-type ATF7IP and MGA in ESCs

For the forced expression of wild-type ATF7IP, cDNA for ATF7IP connected to the IRES-puromycin gene was subcloned into the *piggy*Bac vector carrying the constitutively active chicken actin gene promoter^35^ and introduced into *Atf7ip*-null ESCs together with the *piggy*Bac transposase expression vector, as described previously.^62^ Then, these ESCs were subjected to puromycin selection (1 μg/mL) for 6 days to eliminate untransfected cells. For the forced expression of wild-type MGA, pCAG-HA-IRES-Neomycin expression vector carrying the cDNA for MGA was introduced into MGAΔFAM mutant ESCs. The cells were washed and recovered 24 and 48 h post-transfection, respectively.

#### RNA isolation, reverse transcription, and quantitative PCR (qPCR)

Total RNA from parental ESCs or their derivatives was prepared using RNeasy Mini Kit (QIAGEN Cat# 74104). The prepared RNA was converted to cDNA by reverse transcription using ReverTra Ace (TOYOBO Cat# TRT-101). The cDNA was then used for quantitative PCR (qPCR) by an SYBR Green-based method for *Stag3* gene expression using the oligonucleotides shown in Table S1 qPCRs for *Tex19.1*, *Hormad1*, *Dazl*, *Ddx4*, *Meiosin*, *Mea1*, *Stra8*, and *Gapdh* were performed by a TaqMan-based method using the probes listed in the key resources table of STAR Methods.

#### RNA-seq analyses

Libraries were prepared with total RNA (*N* = 2 for each condition) using TruSeq Stranded Total RNA Library Prep Gold Kit from Illumina (San Diego, CA, USA) with Ribozero Gold (Illumina #MRZG126), in accordance with the manufacturer’s instructions. RNA sequencing was performed on an Illumina NovaSeq 6000 platform with 100 bp paired-end reads with 40–60 million reads for each sample. Sequence reads were trimmed to remove low-quality sequences and adapter sequences using Sickle (version 1.33). Trimmed reads were then mapped to the mm10 reference genome using HISAT2 (version 2.1.0) with the default parameters. The mapped reads were sorted by SAMtools (version 1.10). Read count extraction and normalization were performed using gencode.vM25.Chr_patch_hap1_scaff.annotation.gff3 by StringTie (version 2.1.2). Canonicalization of the gene expression data was performed using edgeR (version 3.34.0) in R-studio (R version 4.1.0).

#### Protein isolation and western blot analysis

Whole-cell extracts were prepared by lysing cells using RIPA buffer (20 mM Tris-HCl pH8.0; 1 mM EDTA; 140 mM NaCl; 1% Triton X-100; 0.1% SDS; 0.1% deoxycholic acid), while nuclear extracts were prepared in accordance with the procedure of Shreiber et al.^63^ in which harvested cells were resuspended in hypotonic buffer (20 mM HEPES-KOH pH7.9; 10 mM NaCl; 1.5 mM MgCl_2_; 0.2 mM EDTA; 20% glycerol; 1% NP-40) by gentle pipetting and were left on ice for 15 min. Micro-test tubes were then centrifuged in a microfuge. Nuclear pellet was resuspended in hypertonic buffer (hypotonic buffer plus 10% volume of 5 M NaCl) and the tube was left on ice for 60 min. Nuclear extract was recovered as supernatant from the tube subjected to centrifugation in a microfuge for 5 min. Histone-enriched protein solutions were prepared in accordance with the procedure of Stein and Mitchell^64^ as follows: Harvested cells (5×10^5^) were resuspended in PBS containing 0.5% Triton X-100, 4 mM sodium butyrate, and protease inhibitor cocktail. After lysis by gentle stirring on ice for 10 min, nuclei were recovered by centrifugation in a microfuge for 10 min. After resuspension in fresh buffer and re-centrifugation, recovered nuclei were left on ice for 1 h after resuspension in 125 μL of 0.4 N HCl solution. After centrifugation in a microfuge, supernatant was recovered as histone-enriched protein solutions and treated with 1.25 mL of acetone for precipitation. The micro-test tube was subjected to centrifugation for 10 min after being left at −20°C overnight and then proteins recovered as a pellet were dissolved with an appropriate volume of H_2_O. For western blot analyses, protein solutions were initially subjected to heat treatment (100°C) for 3 min after mixing with 2× SDS-PAGE sample buffer (0.1 M Tris-HCl pH 6.8; 4% SDS, 0.2 M DTT; 0.2% bromophenol blue; 20% glycerol) (1:1). Subsequently, protein samples were subjected to size fractionation on SDS-polyacrylamide gels and then electrically transferred to a PVDF membrane. The protein-bound membrane was blocked with 5% fat-free milk at room temperature for at least 2 h and then incubated with primary antibody at 4°C overnight. After three rounds of washing with PBS containing 0.1% Tween 20, the membrane was incubated with horseradish peroxidase-conjugated goat anti-rabbit IgG (1:2000: Cell Signaling Technology #7076) or goat anti-mouse IgG (1:2000: Cell Signaling Technology #7074) secondary antibody at room temperature for 1 h. After washing with PBS containing 0.1% Tween 20 three times, protein signals were visualized with enhanced chemiluminescence detection reagents (MERCK Cat# RPN2109).

#### Coimmunoprecipitation analysis

For coimmunoprecipitation analyses, control ESCs and those subjected to specific genetic modification (at the *Atf7ip*, *Zmym2*, or *Mga* locus) were suspended in a buffer (10 mM Tris-HCl pH8.0, 1 mM EDTA, 140 mM NaCl, 1% NP-40). After centrifugation, the supernatant was recovered and incubated with anti-MGA antibody that had been preincubated with Dynabeads with anti-rabbit IgG antibody for 2 h at 4°C in either the presence or the absence of a mixture of DNase and RNase. After incubation overnight at 4°C, bead-bound proteins were recovered by brief centrifugation, extensively washed three times with PBS, and then eluted with 1× SDS-PAGE sample buffer. Proteins were then subjected to western blot analysis as described above except for the use of TrueBlot ULTRA goat anti-rabbit Ig HRP (ROCKLAND, 18-8816-31) as the secondary antibody. Exactly the same procedure was conducted for coimmunoprecipitation analyses using HEK293FT cells expressing HA- and/or Flag-tagged exogenous proteins, except for the use of Dynabeads with anti-mouse IgG antibody for immunoprecipitation and TrueBlot ULTRA goat anti-mouse Ig HRP (ROCKLAND, 18-8817-31) as the secondary antibody.

#### Chromatin immunoprecipitation (ChIP)-qPCR analyses

Chromatin immunoprecipitation (ChIP)-qPCR analyses were conducted using parental ESCs and their derivatives. Cells (2×10^6^) were first treated with 1% formaldehyde in PBS for 10 min at room temperature before quenching the crosslinks with glycine solution. Then, cells were washed with PBS, collected, and resuspended in hypotonic buffer (10 mM Tris-HCl pH 8.1, 10 mM NaCl, 1.5 mM MgCl_2_, and 0.5% Igepal-CA630). After the addition of protease inhibitor cocktail and PMSF solution, cells were placed on ice for 15 min with gentle pipetting (10 times every 5 min) for cell lysis. After centrifugation in a microfuge for 5 min and removal of the supernatant, the nuclear pellet was resuspended in nuclear lysis buffer (50 mM Tris-HCl pH 8.1, 5 mM EDTA, and 1.0% SDS) and then mixed with protease inhibitor cocktail and PMSF solution as above. Subsequently, the lysate was passed through a needle (23-gauge) 20 times and then subjected to sonication for 30 min (ON for 30 s and OFF for 30 s for each cycle), followed by recovery of the lysate after 10 min of centrifugation. The lysate (50 μL) was mixed with 450 μL of ChIP buffer (16.7 mM Tris-HCl, 1.1% Triton X-100, 0.01% SDS, and 167 mM NaCl) containing protease inhibitor cocktail and PMSF, 20 μL of protein A magnetic beads (Millipore Cat# 16–661), and 1.5 μL of a specific antibody and then incubated at 4°C overnight with gentle rotation. After removal of the supernatant using a magnetic stand, the beads were washed with buffers in the following order: wash buffer I (0.1% SDS, 1% Triton X-100, 2 mM EDTA, 20 mM Tris-HCl pH8.1, and 20 mM NaCl), wash buffer II (the same as wash buffer I except that the NaCl concentration was 500 mM), wash buffer III (1% Igepal-CA630, 1% deoxycholate, 1 mM EDTA, 10 mM Tris-HCl pH8.1, and 250 mM LiCl), and TE buffer (10 mM Tris HCl pH8.0 and 1 mM EDTA). Subsequently, immunoprecipitated genomic DNA was eluted from beads with elution buffer (1% SDS and 0.1 M NaHCO_3_) in the presence of proteinase K. Tubes were then treated sequentially at 62°C and 95°C for 4 h and 10 min, respectively. Genomic DNA recovered as a supernatant was purified using a QIAquick PCR Purification Kit (Cat# 28104) and used for SYBR Green-based qPCR. Specific primers used in these analyses are listed in Table S1.

#### ChIP-seq analysis

The ChIP reaction was performed as described above except for the use of more cells (1×10^7^) for each reaction. Analysis of the data from the ChIP-seq experiment was conducted essentially as described previously.^22^ Raw data of sequence reads were subjected to filtration to check their quality using Sickle (version 1.33, parameters -q 20 -l 20) and subsequently mapped to the mouse genome (mm10 assembly) using bowtie2 (version 2.4.1) software. The resulting SAM files were converted to BAM format with the aid of SAMtools (version 1.1.0) and then the obtained BAM files were subjected to the PEAK CALL procedure using MACS2 (version 2.2.7.1) software with the parameters -q 1e10 -c for each IgG file. Peaks identified by the procedure were subjected to the Two Nearest Genes-association rules (within 5 kb) of the Genomic Regions Enrichment of Annotations Tool (http://bejerano.stanford.edu/great/public/html/splash.php). BigWig files were generated using the software bamCoverage or bamCompare from deepTools 3.4.3. Heatmaps and metaplots around the Mga binding sites were generated using computeMatrix and plotHeatmap software from deeptools 3.4.3. Enrichment scores around the Mga binding sites were generated using multiBigwigSummary software from deeptools 3.4.3.

#### RNA-seq analyses

Libraries were prepared with total RNA (N=2 for each condition) using TruSeq Stranded Total RNA Library Prep Gold Kit from Illumina (San Diego, CA, USA) with Ribozero Gold (Illumina #MRZG126), in accordance with the manufacturer’s instructions. RNA sequencing was performed on an Illumina NovaSeq 6000 platform with 100 bp paired-end reads, with 40–60 million reads for each sample. Sequence reads were trimmed to remove low-quality sequences and adapter sequences using Sickle (version 1.33). Trimmed reads were then mapped to the mm10 reference genome using HISAT2 (version 2.1.0) with the default parameters. The mapped reads were sorted by SAMtools (version 1.10). Read count extraction and normalization were performed using gencode.vM25.Chr_patch_hap1_scaff.annotation.gff3 by StringTie (version 2.1.2). Canonicalization of the gene expression data was performed using edgeR (version 3.34.0) in R-studio (R version 4.1.0).

#### TetO array analysis

DNA encoding the carboxy-terminal region of MGA (amino acids 2445–2844) or its derivative lacking the FAM motif was subcloned into the region immediately downstream of the Flag-tagged TetR coding sequence in pCAGFS2TETR. These plasmids were individually introduced into the TetO element-containing ESCs (E14TetIN ESCs) and then stable ESC clones expressing fusion proteins comprising TetR and the carboxy-terminal portion of MGA or its derivative were selected by western blot analysis using anti-Flag M2 antibody. These ESCs were then used for ChIP to examine the levels of PCGF6, SETDB1, and repressive histone marks (H2AK119ub, H3K27me3, H3K9me2, and H3K9m3) as well as fusion proteins by themselves, as described in the section on Chromatin immunoprecipitation (ChIP)–qPCR analyses. The primers used in the analysis were described by Blackledge et al.^37^

### Quantification and statistical analysis

The data from qPCR analyses are displayed as mean ± SD and were derived from three biological replicates. For statistical significance assessment, one-way ANOVA followed by Bonferroni’s Post-Hoc test was conducted for data shown in Figures 4F and S14, while hypergeometric test was performed to assess the significance of the overlap between two gene groups. For all of other data, two-tailed unpaired Welch’s t-test was used.

## Supplemental information

Document S1. Figures S1–S16 and Tables S1 and S3

Table S2. Gene lists categrized into each cluster
