# Supplementary material for: MGA directly recruits SETDB1/ATF7IP for histone H3K9me3 mark on meiosis-related genes in mouse embryonic stem cells
Source: iScience. 2025 Jul 5;28(8):113059. doi: 10.1016/j.isci.2025.113059 (PMC12303078; doi:10.1016/j.isci.2025.113059)
Supplement: Document S1. Figures S1–S16 and Tables S1 and S3 [file mmc1.pdf]

iScience, Volume *28*

Supplemental information

MGA directly recruits SETDB1/ATF7IP for histone H3K9me3 mark on meiosis-related genes in mouse embryonic stem cells

Kousuke Uranishi, Masataka Hirasaki, Masazumi Nishimoto, Robert J. Klose, Akihiko Okuda, and Ayumu Suzuki

A

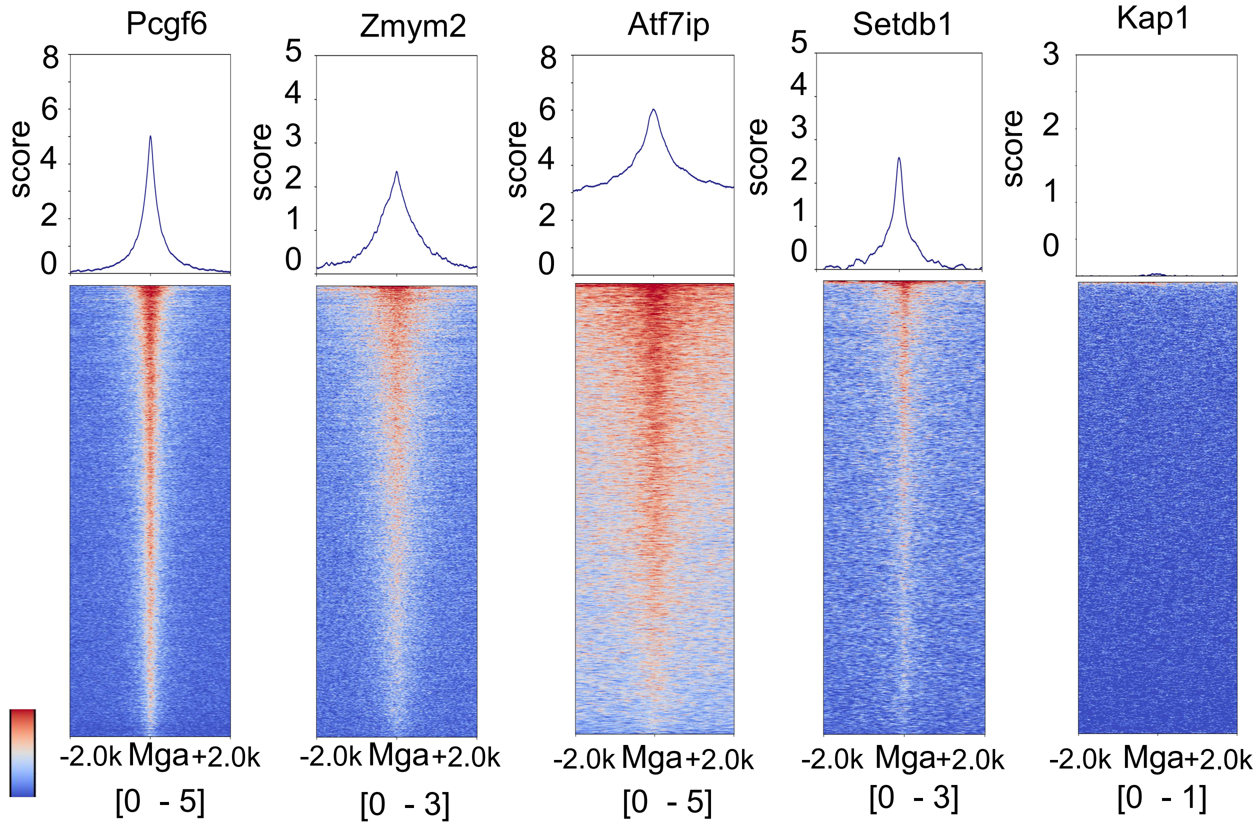

B

Genomic sites	Mga	Pcgf6	Atf7ip	Zmym2	Setdb1
4,900	●				
600	●	●			
1	●	●			●
83	●	●		●	
7	●	●		●	●
51	●	●	●		
2	●	●	●		●
33	●	●	●	●	
21	●	●	●	●	●
4	●				●
222	●			●	
4	●			●	●
72	●		●		
1	●		●		●
21	●		●	●	
6	●		●	●	●

Figure S1. Heatmap analyses of ChIP-sequence data for PCGF6 and factors related to H3K9me3 modification on genomic MGA binding sites (A) Genomic MGA binding peaks were ranked from the strongest to weakest signal for each factor. (B) A table showing the overlap among PCGF6, ATF7IP, ZMYM2, and SETDB1 on MGA-bound genomic sites.

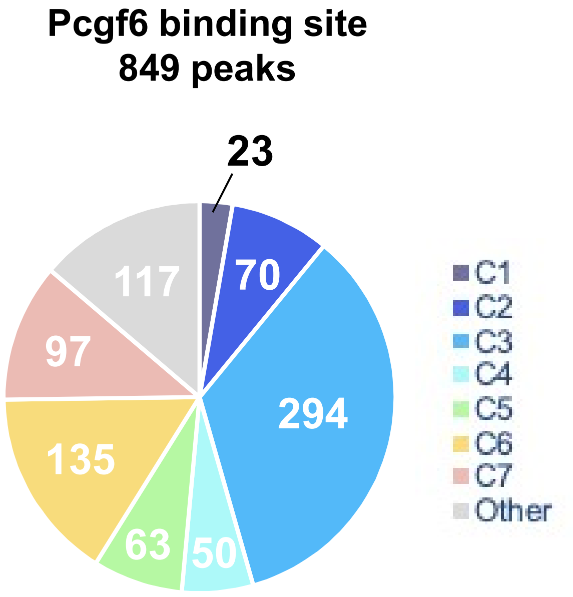

Figure S2. Pie chart showing the distribution of genomic PCGF6-bound sites among seven cluster gene sets. A total of 117 of 849 sites are not included in any cluster.

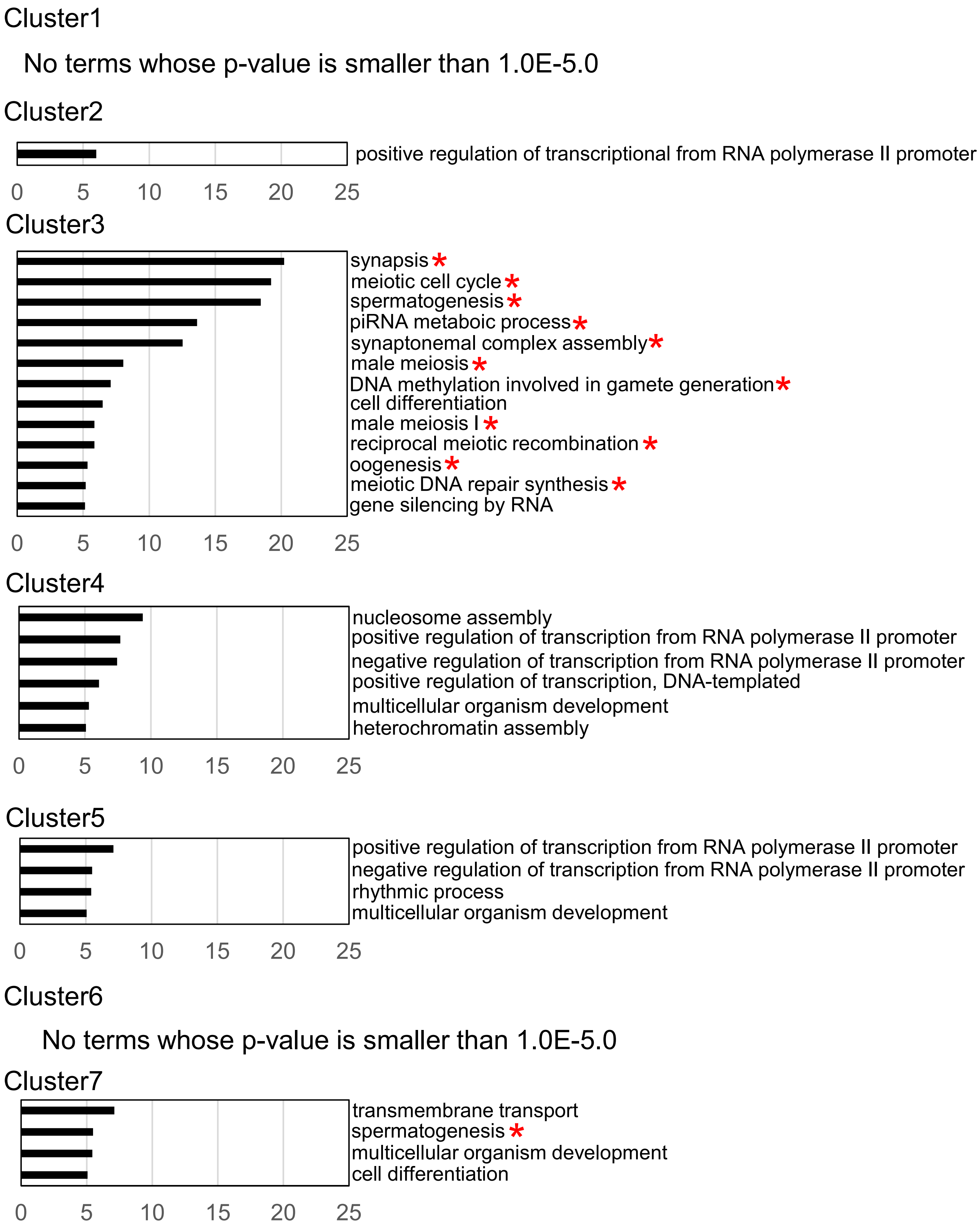

Figure S3. GO analyses of seven MGA binding site-containing gene sets Terms whose *p*-value is less than 1.0×10^{-5} are shown.

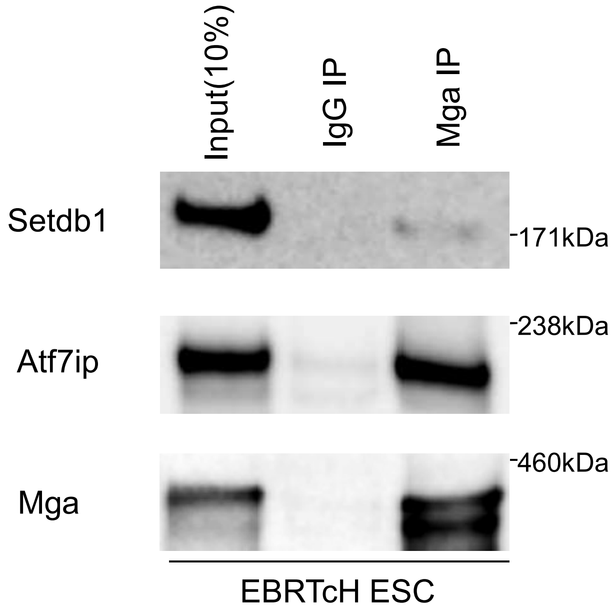

Figure S4. Coimmunoprecipitation analyses examining whether either DNA or RNA is required for the interaction between MGA and ATF7IP/SETDB1 complex Coimmunoprecipitation analyses were performed with anti-MGA antibody using nuclear extracts from wild-type ESCs as in Figure 1D, but the reactions were carried out in the presence of DNase (50 units) and RNase (20 μg). IgG from non-immune rabbits was used as a negative control for immunoprecipitation.

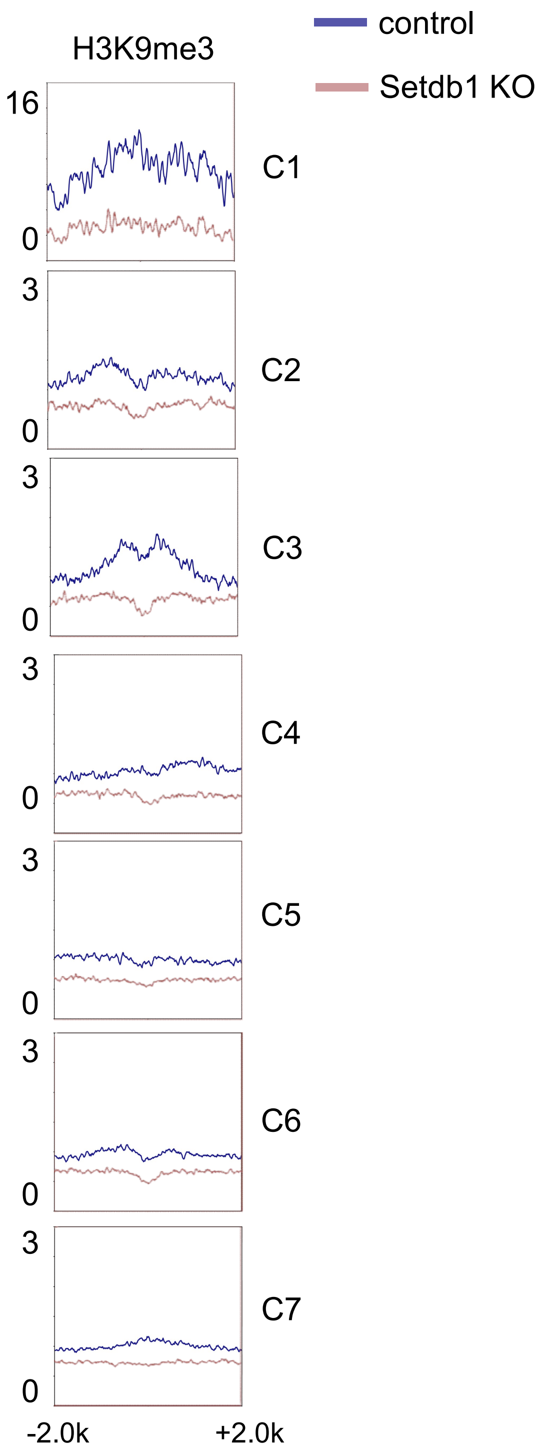

Figure S5. Uniform dependence on SETDB1 for maintaining H3K9me3 levels among seven MGA binding site-containing gene cluster sets The H3K9me3 levels of seven cluster gene sets were individually compared between wild-type and *Setdb1*-null ESCs.

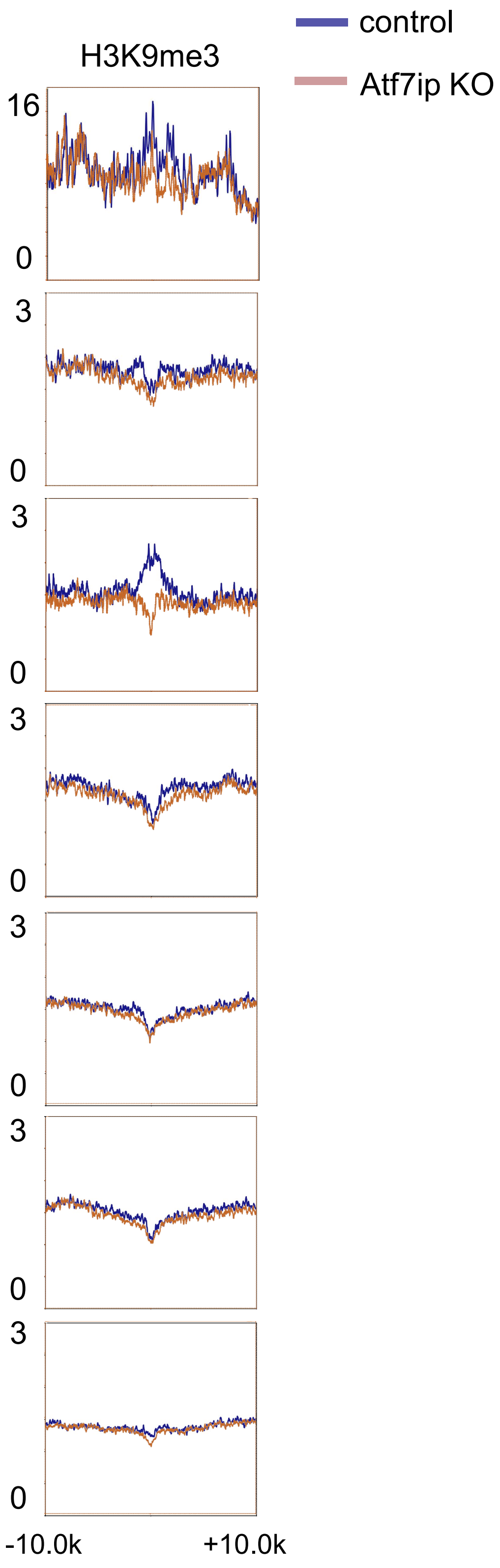

Figure S6. Metagene plots for comparing the effect of *Atf7ip* gene disruption on maintaining H3K9me3 levels among seven MGA binding site-containing gene cluster sets These metagene plots correspond to broader images of those shown in Figure 2A.

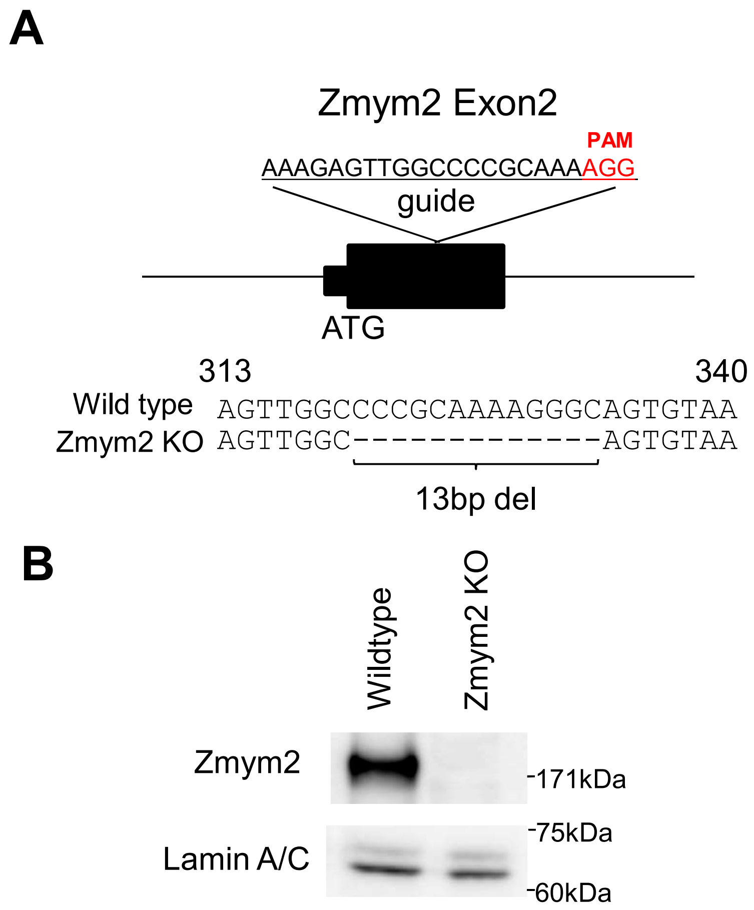

Figure S7. Generation of *Zmym2*-null ESCs (A) Schematic representation of the strategy for generating *Zmym2*-null ESCs. Exon 2 bearing a translation-initiating methionine codon was genetically modified using the CRISPR–Cas9 system with the indicated oligonucleotide as a guide sequence. After the procedure, ESCs possessing the out-of-frame mutation (13-bp deletion) within exon 2 were selected and used for subsequent analyses. (B) Western blot analyses of ZMYM2 using nuclear extracts from wild-type and *Zmym2*-null ESCs. LaminA/C protein level was used as an internal control.

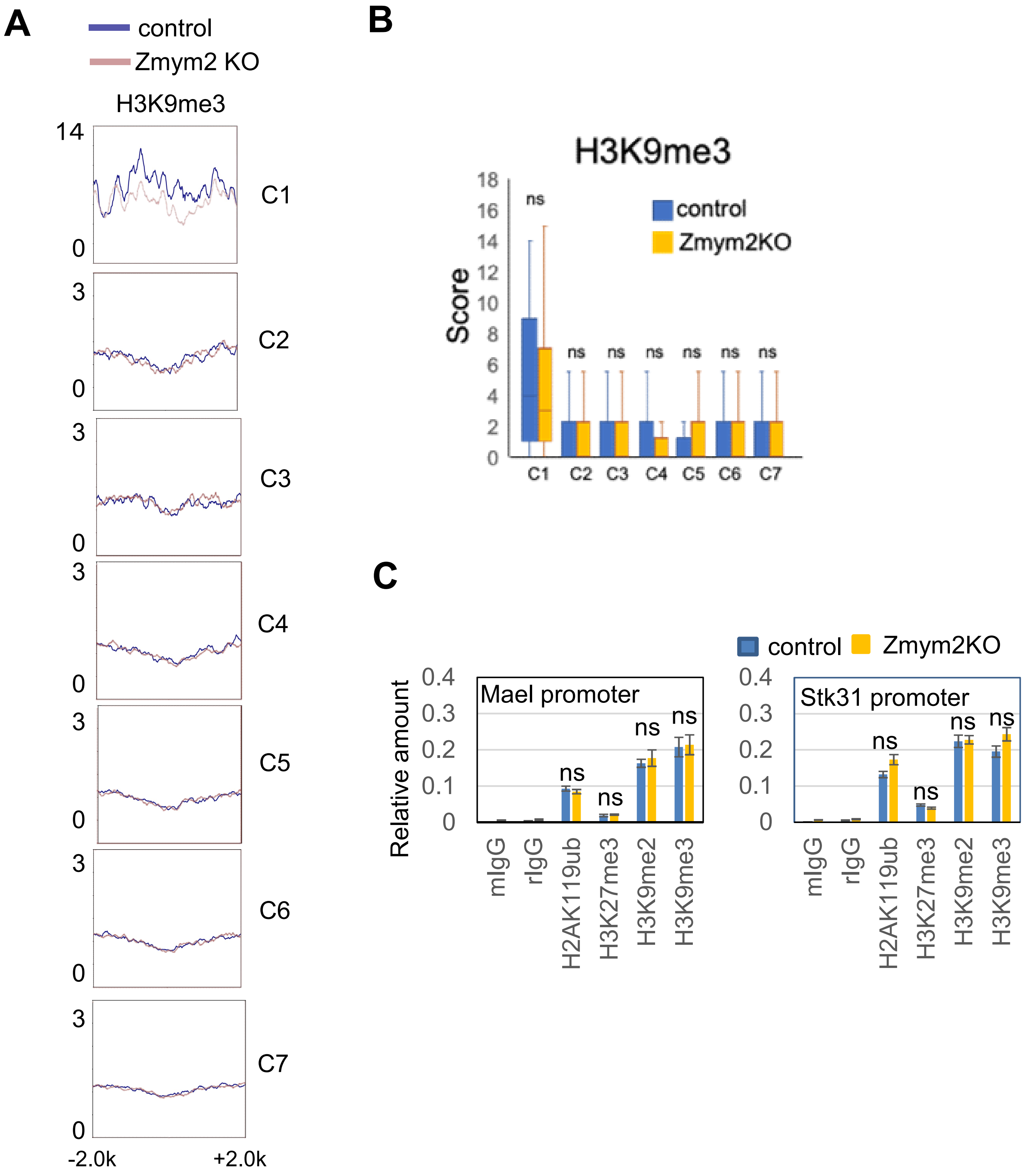

Figure S8. Examination of the effect of *Zmym2* gene disruption on H3K9me3 and other histone modifications (A) Metagene plots for comparing the effect of *Zmym2* gene disruption on maintaining H3K9me3 levels among seven MGA binding site-containing gene cluster sets. (B) Quantitative analysis of the difference in H3K9me3 levels due to Zmym2 gene disruption in each gene cluster using a box plot. Statistical significance was assessed using two-unpaired Welch's t-test. ns, $p>0.05$. (C) ChIP-qPCR analyses of H2AK119ub, H3K27me3, H3K9me2, and H3K9me3 on *Mael* and *Stk31* gene promoters using wild-type and *Zmym2*-null ESCs. Data represent mean ± SD of three independent experiments. Two-tailed unpaired Welch's t-test was conducted to examine statistical significance. ns, $p>0.05$.

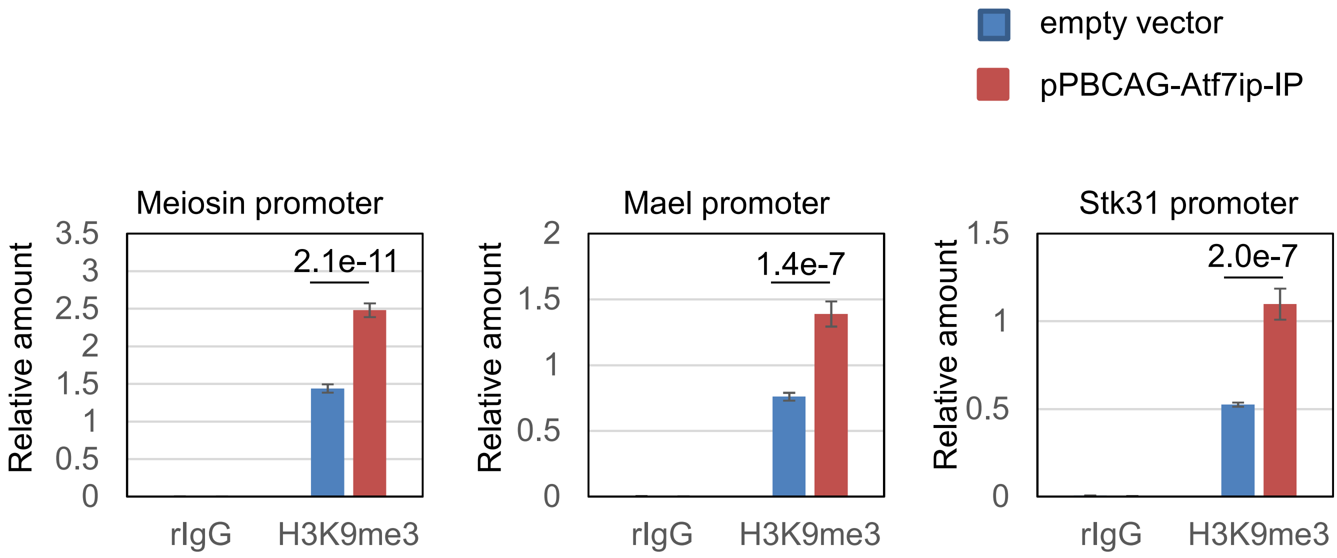

Figure S9. Forced expression of *Atf7ip* gene in *Atf7ip*-null ESCs was accompanied by the elevation of H3K9me3 levels of meiosis-related genes *Atf7ip*-null ESCs expressing exogenous ATF7IP stably via the *piggy*Bac system were used for ChIP-qPCR analyses of H3K9me3 on *Meiosin*, *Mael*, and *Stk31* gene promoters. *Atf7ip*-null ESCs carrying the *piggyBac* empty vector were used as reference cells. Data represent mean ± SD of three independent experiments. Two tailed unpaired Welch's t-test was conducted to examine statistical significance.

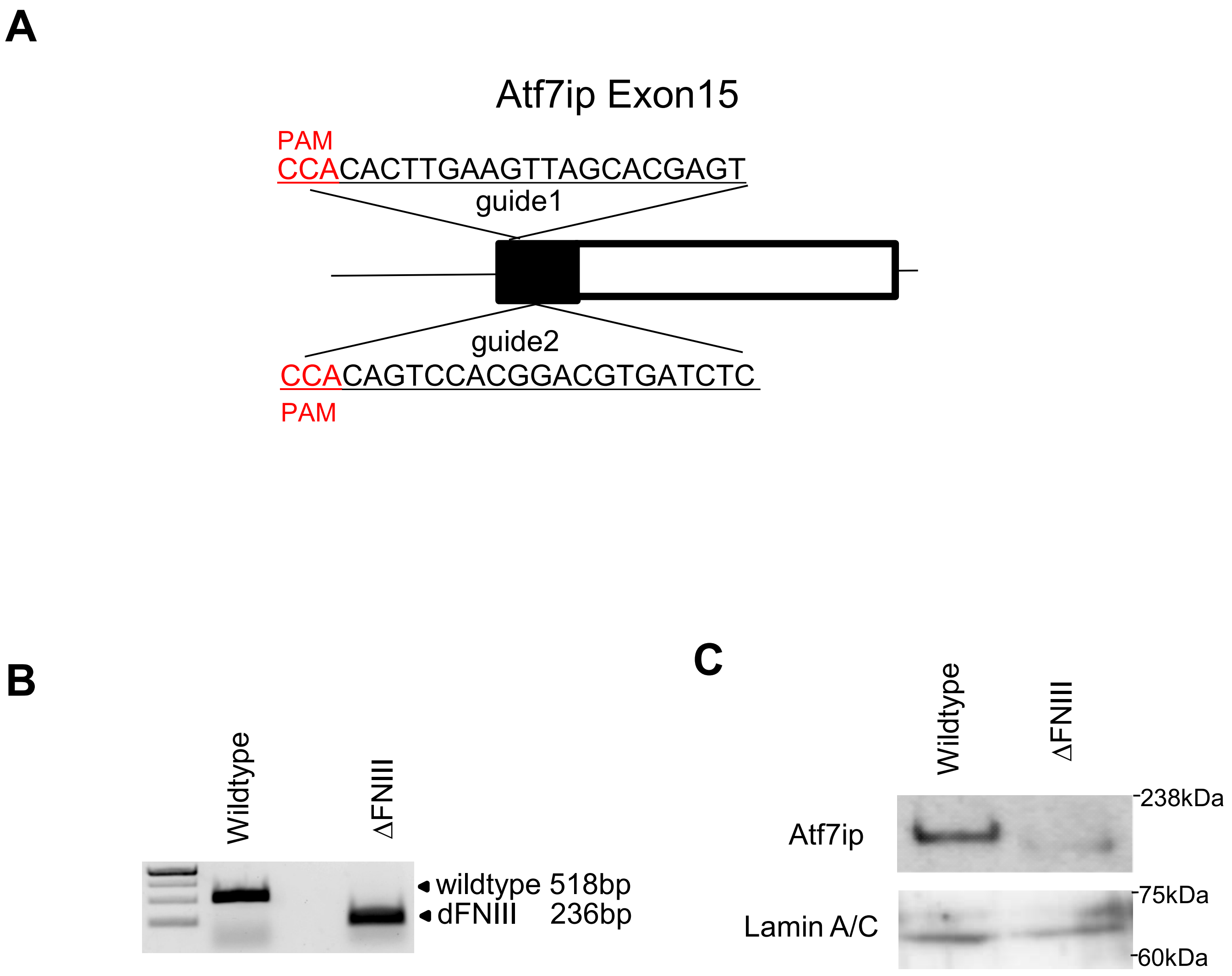

Figure S10. Generation of ESCs producing ATF7IP lacking FNIII domain (A) Schematic representation of the strategy for deleting the region encoding the FNIII domain of ATF7IP in ESCs using the CRISPR–Cas9 system with the indicated oligonucleotides. Solid and open rectangles represent coding and noncoding regions of exon 15 of the *Atf7ip* gene. (B) PCR analyses for confirming the ESC clones in which genetic manipulation had been achieved as expected. Exact deletion of the FNIII-encoding genomic region was confirmed by DNA sequencing. (C) Western blot analyses of ATF7IP using nuclear extracts from wild-type ESCs and those producing ATF7IP lacking its FNIII domain. LaminA/C protein level was used as an internal control of nuclear extract.

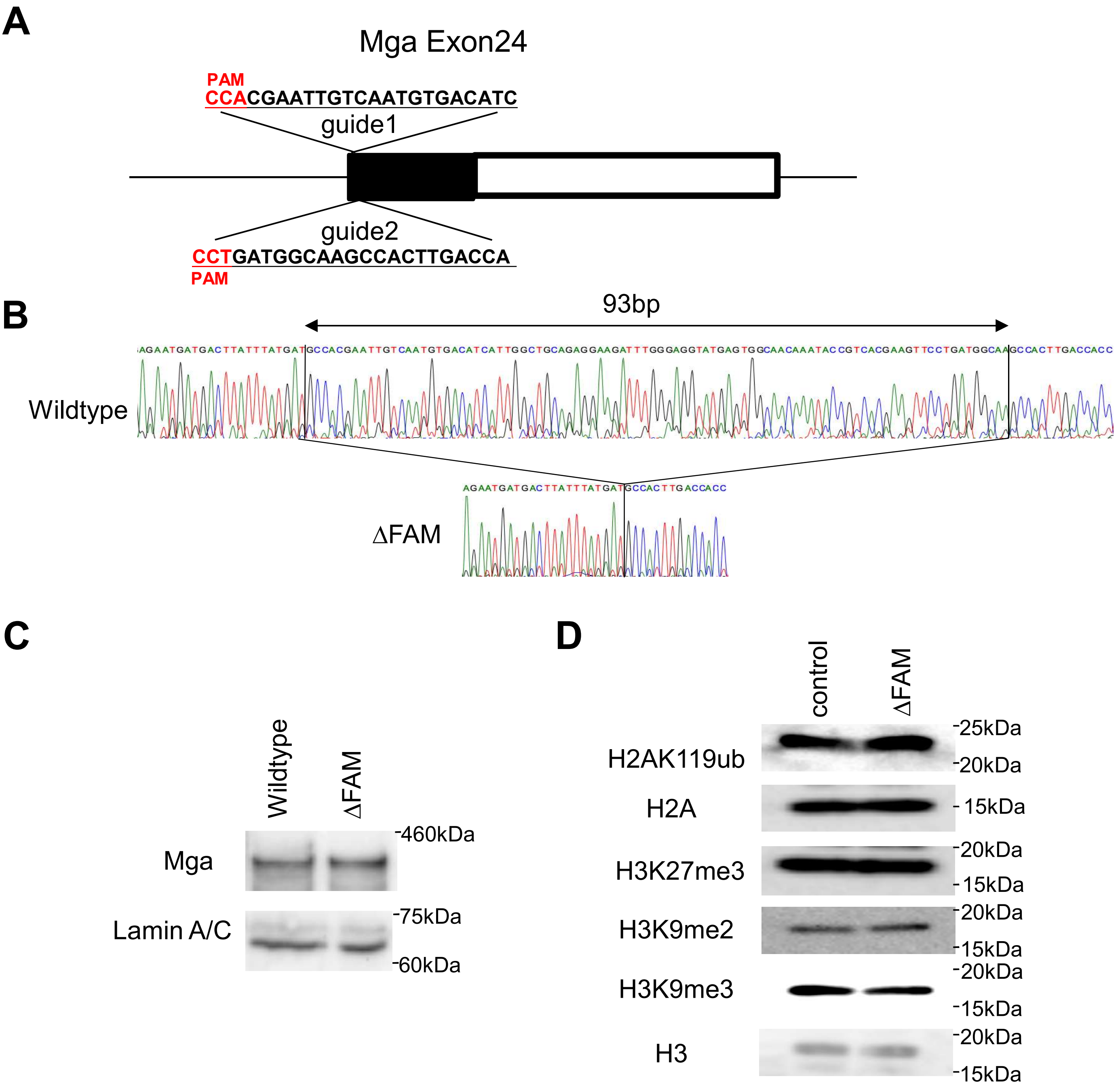

Figure S11. Generation of ESCs producing MGA lacking FAM domain (A) Schematic representation of the strategy for deleting the region that encodes the FAM domain of MGA in ESCs using the CRISPR–Cas9 system with the indicated oligonucleotides. Solid and open rectangles represent coding and noncoding regions of exon 24 of the *Mga* gene. (B) ESCs yielding MGA lacking its FAM domain due to in-frame deletion (93 bp) were selected and used for subsequent analyses. (C) Western blot analyses of MGA using nuclear extracts from wild-type ESCs and those producing MGA that lacks its FAM domain. LaminA/C protein level was used as an internal control of the nuclear extract. (D) Western blot analyses to compare the total amounts of histone H2A and H3 and their epigenetically modified derivatives between wild-type ESCs and those producing MGA lacking its FAM domain.

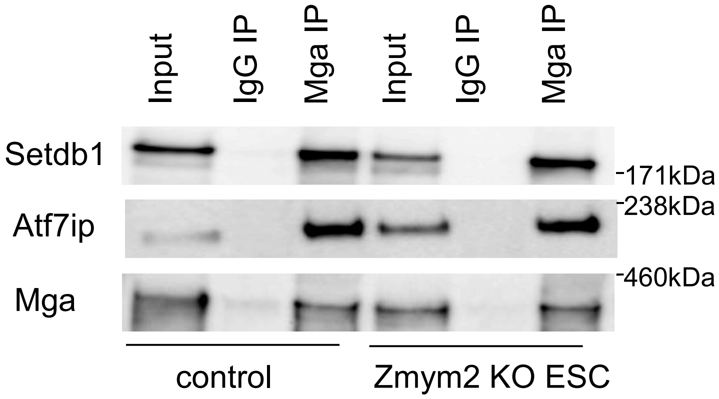

Figure S12. Examination of effect of *Zmym2* gene disruption on interaction of MGA with SETDB1 and ATF7IP Coimmunoprecipitation analyses were performed with anti-MGA antibody using nuclear extracts from wild-type and *Zmym2*-null ESCs and then the amounts of immunoprecipitated SETDB1 and ATF7IP were compared between wild-type and mutant ESCs.

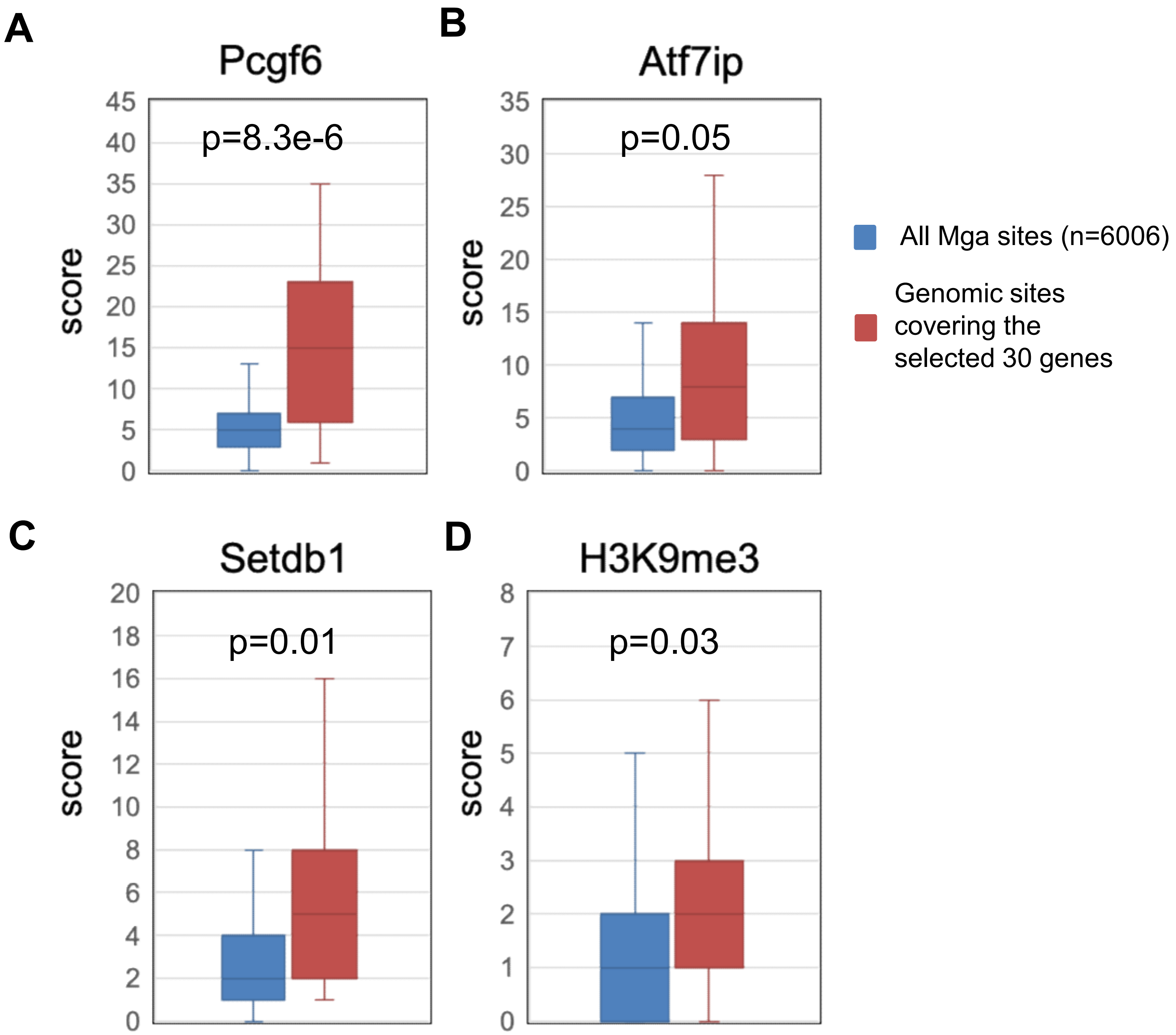

Figure S13. Boxplots showing the levels of PCGF6, ATF7IP, SETDB1 and H3K9me3 of 30 MGA-bound genes that were derepressed in ΔFAM MGA mutant ESCs Thirty-five genomic sites covering the selected 30 genes in Figure 4B as genes that bind to MGA as well as those activated in ΔFAM MGA mutant ESCs were compared to all-MGA bound genomic sites with respect to accumulation levels of PCGF6, ATF7IP, SETDB1 and H3K9me3. Statistical significance was assessed using two-unpaired Welch's t-test. ns, $p>0.05$.

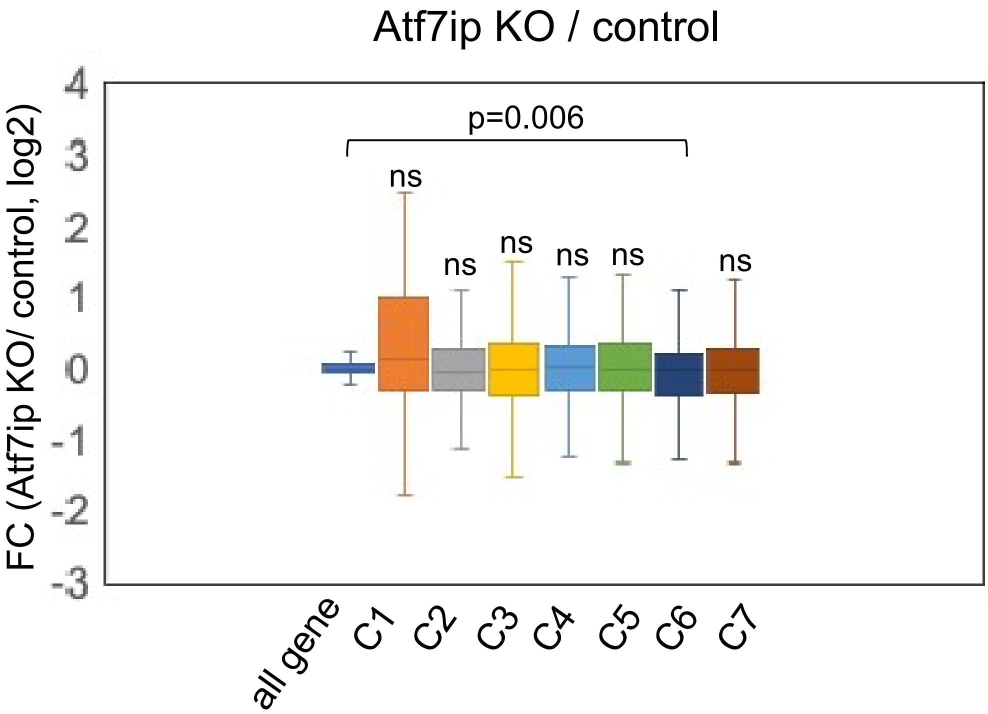

FC(log2)	All	C1	C2	C3	C4	C5	C6	C7
Average	0.01	0.32	-0.02	0.05	0.02	0.03	-0.07	-0.01
Median	0.00	0.13	-0.03	0.00	0.03	0.00	-0.01	0.00

Figure S14. Box plots for examining the effect of *Atf7ip* gene disruption on transcriptional alterations of all genes and seven gene clusters. Data for transcriptional alteration due to *Atf7ip* gene disruption from each gene cluster were compared to those from all annotated coding and noncoding genes (n=55,492). Statistical significance was determined using one-way ANOVA followed by Bonferroni's Post-Hoc test. ns, $p>0.05$.

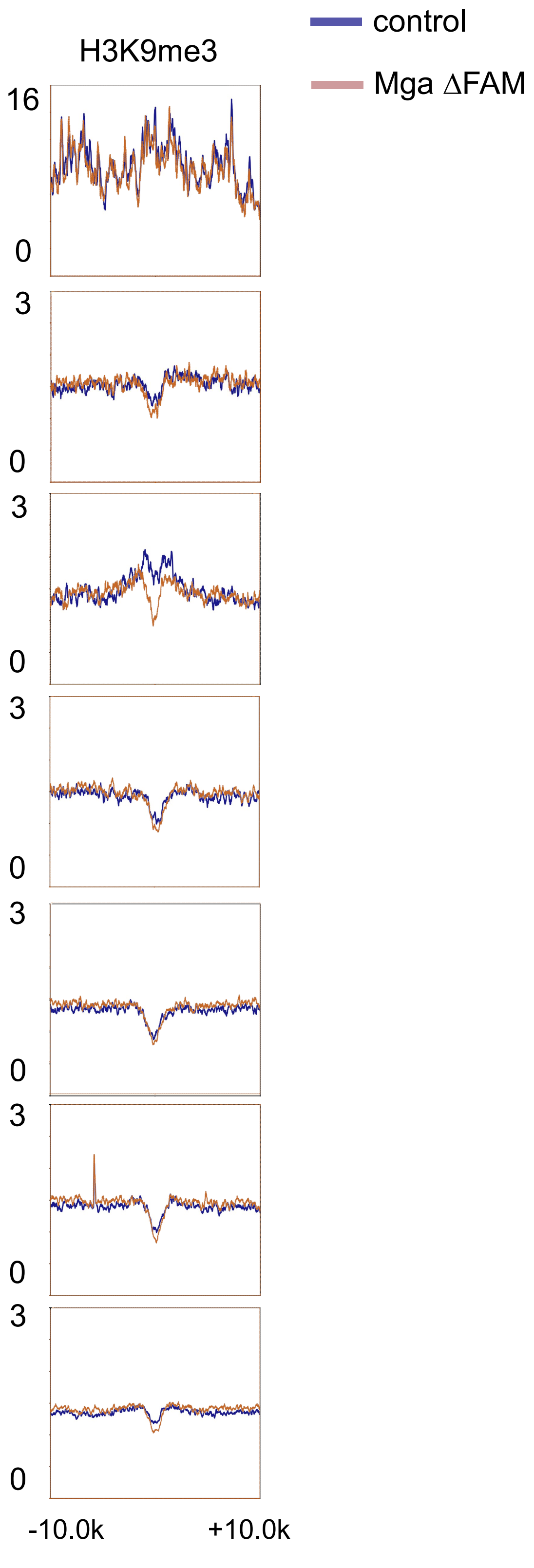

Figure S15. Metagene plots for comparing the effect of removal of FAM from MGA on maintaining H3K9me3 levels among seven MGA binding site-containing gene cluster sets These Metagene plots correspond to broader images of those shown in Figure 5A.

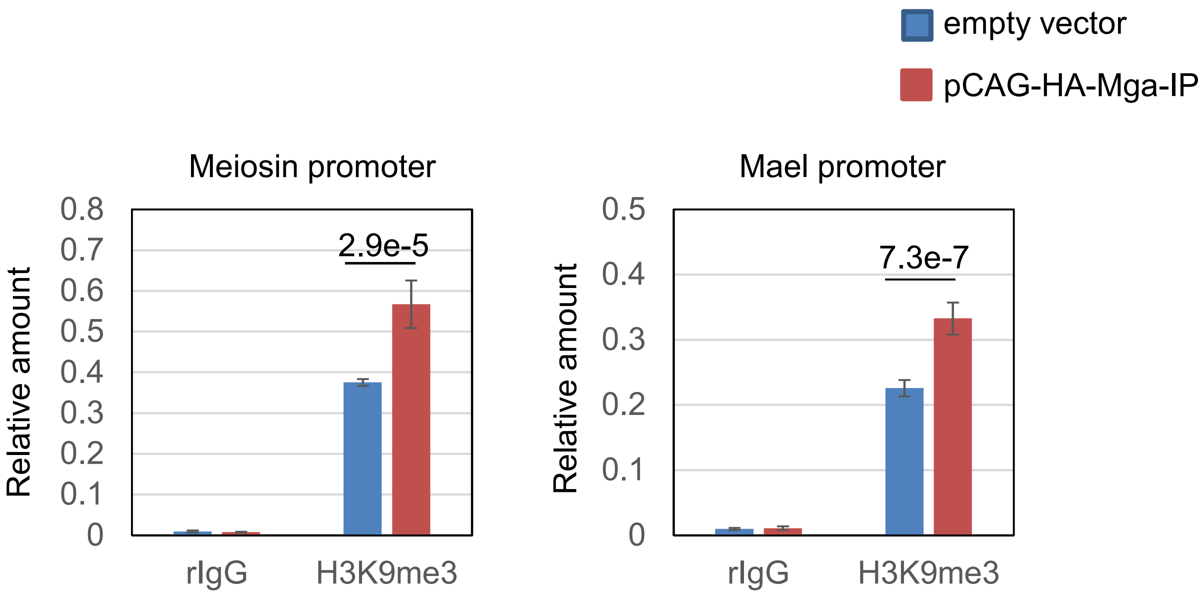

Figure S16. Forced expression of the cDNA encoding wild-type MGA in ΔFAM MGA mutant ESCs was accompanied by the elevation of H3K9me3 levels of meiosis-related genes ΔFAM MGA mutant ESCs in which *Mga* expression vector or empty vector was transiently introduced were used for ChIP-qPCR analyses of H3K9me3 on *Meiosin* and *Mael* gene promoters. Data represent mean ± SD of three independent experiments. Two-tailed unpaired Welch's t-test was conducted to examine statistical significance.

Table S1

Sequences of oligonucleotides

Oligonucleotides for genetic manipulation by CRISPR/Cas9 in ESCs

Oligonucleotides for targeting immediately upstream (upper set) and downstream (lower set) of the region corresponding to FAM to generate ESCs expressing ΔFAM mutant of MGA

5'-caccgATGTCACATTGACAATTCG-3'* 5'-aaacCGAATTGTCAATGTGACATc-3'

5'-caccgTGGTCAAGTGGCTTGCCATC-3' 5'-aaacGATGGCAAGCCACTTGACCAc-3'

Oligonucleotides for targeting immediately upstream (upper set) and downstream (lower set) of the region corresponding to FNIII to generate ESCs expressing ΔFNIII mutant of ATF7IP

5'-caccgAGATCACGTCCGTGGACTG-3'* 5'-aaacCACTTGAAGTTAGCACGAGTc-3'

5'-caccGAGATCACGTCCGTGGACTG-3' 5'-aaacCAGTCCACGGACGTGATCTC-3'

Genotyping

Mga Intron 23 – Exon 24

5'-CAGAGCTGAGGACCAAACCCAG-3' 5'- TCCATGTTAGTCAGCAGCTCACTG-3'

Zmym2 Exon 2

5'-GTGTCATAGCCATATCTCTGCAGATG-3' 5'-CTGCCCTATCAATTTATCTGCCTGG-3'

Atf7ip Exon 15

5'-CTCTGCATCCAGCGCCCTTA -3' 5'-GTCCGGGAGATCTTGCACAAGG-3'

Quantitative PCR for *Stag3* gene expression

5'-GCAACCAGCTGATGCGACTC-3' 5'-GCGCTCACTGGTGGAAGAGTAG-3'

ChIP- and MeDIP-qPCRs

Mael (for ChIP- and MeDIP-qPCRs)

5'-GGTGGAAATCCAGTTTCAGGCTGTT-3' 5'- ACTTGGCCAATCAGGGTGCAG-3'

Stag31 (for ChIP-qPCR)

5'- GCAACCAGCTGATGCGACTC -3' 5'- GCGCTCACTGGTGGAAGAGTAG -3'

Meiosin (for ChIP- and MeDIP-qPCRs)

5'-AGCAGAAGTCGAGGCTTAACGC-3' 5'-TGACTCTGACGACTCCTCACCAT-3'

---

*Sequences of forward and reverse primers are provided in left- and right-hand sides, respectively.

Table S3

(A)

	activated gene	down-regulated genes
cluster 1	**Fkbp6**	
cluster 2	**Dmrt1**, Tasor2	
cluster 3	8030474K03Rik, Dazl, Ddx4, Fam178b, Gpat2, Hormad1, Hormad2, Mael, Meiosin, Mov10l1, **Prr19**, Rnf17, Sec1, Slc25a31, Stag3, Stk31, Syce1, Sycp3, **Taf7l**, Tex19.1, **Zcwpw1**	
cluster 4	**Zcwpw1**	
cluster 5	**Dmrt1**, **Taf7l**, Tdrd12, Zbtb32	
cluster 6	Tuba3a	Slc12a8
cluster 7	**Fkbp6**, **Prr19**, Rhox13, Rpl10l, Tex11	
non-clustered	Atp5e, Bmf, Dnajb6, Fbrsl1, Gm13610, Gm15529, Gm28625, Gm44346, Gm44367, Gm44454, Gm45660, Gm4779, Gm50449, Mapkbp1, Mir290b, Mir677, Pkp4, Rhox5, Rpl30	Dmtn, Eif4e, Esd, Fanca, Gm6061, Gm20632, Gm44394, Gm47311, Krt17, Lcmt1, Miip, Pmel, Rabep2, Rpl30-ps3, Rpl30-ps8, S100a13, Snord72, Ube2d3, Zfp229

Genes included in multiple clusters are indicated by bold letter

(B)

GO ID	GO term	p-value	Count	Genes
GO:0007283	spermatogenesis	2.59E-16	14 / 508*	DDX4, DMRT1, RPL10L, TEX19.1, STK31, DAZL, ZCWPW1, TDRD12, MAEL, MOV10L1, SYCP3, HORMAD1, SLC25A31, FKBP6
GO:0034587	piRNA processing	2.09E-14	7 / 21	STK31, DDX4, TDRD12, MAEL, MOV10L1, GPAT2, FKBP6
GO:0007140	male meiotic nuclear division	2.48E-10	6 / 38	DDX4, TEX11, TDRD12, MAEL, MOV10L1, TEX19.1
GO:0007129	homologous chromosome pairing at meiosis	4.74E-10	6 / 43	ZCWPW1, STAG3, MAEL, SYCP3, HORMAD1, TEX19.1
GO:0141196	transposable element silencing by piRNA-mediated DNA methylation	1.48E-09	5 / 17	DDX4, TDRD12, MAEL, MOV10L1, FKBP6
GO:0051321	meiotic cell cycle	3.10E-08	7 / 203	PRR19, TDRD12, MAEL, HORMAD2, SYCP3, HORMAD1, FKBP6
GO:0030154	cell differentiation	1.17E-07	9 / 660	DAZL, ZCWPW1, DDX4, RPL10L, TDRD12, MOV10L1, SLC25A31, TEX19.1, FKBP6
GO:0007130	synaptonemal complex assembly	2.15E-06	4 / 27	SYCE1, TEX11, SYCP3, HORMAD1
GO:0007141	male meiosis I	3.29E-06	4 / 31	DDX4, RPL10L, MOV10L1, SLC25A31

* Numerator and denominator numbers represent the number of the genes included among 30 genes tested and total number of genes constituting each GO term, respectively.
